# Efficacy and safety of IL-17, IL-12/23, and IL-23 inhibitors for psoriatic arthritis: a network meta-analysis of randomized controlled trials

**DOI:** 10.3389/fimmu.2025.1654343

**Published:** 2025-09-18

**Authors:** Shan Gao, Xingxing Xie, Ling Fan, Linyuan Yu

**Affiliations:** ^1^ Department of Pharmacy, Chengdu Second People’s Hospital, Chengdu, Sichuan, China; ^2^ Department of Pharmacy, Yaan People’s Hospital, Yaan, Sichuan, China; ^3^ Department of Good Clinical Practice, Yaan People’s Hospital, Yaan, Sichuan, China; ^4^ Department of Pharmacy, Sichuan Provincial Maternity and Child Health Care Hospital and The Affiliated Women’s and Children’s Hospital of Chengdu Medical College, Chengdu, Sichuan, China

**Keywords:** psoriatic arthritis, IL-17 inhibitors, IL-12/23 inhibitors, IL-23 inhibitors, network meta-analysis

## Abstract

**Background:**

Psoriatic arthritis (PsA) is a chronic inflammatory disease that impacts both the skin and joints. Currently, interleukin (IL)-17, IL-12/23, and IL-23 inhibitors have become integral components of PsA treatment regimens. Nevertheless, the comparative effectiveness of these IL-targeted therapies remains a subject of ongoing debate. This study employs a network meta-analysis (NMA) approach to systematically evaluate the therapeutic efficacy and safety profiles of various IL-17, IL-12/23, and IL-23 inhibitors.

**Methods:**

We searched PubMed, Web of Science, and Embase for randomized controlled trials (RCTs) to identify eligible research articles. This NMA was implemented by Stata 14.0 software, with odds ratios (ORs) and 95% confidence intervals (CIs) serving as effect and safety measures to evaluate clinical efficacy and safety profiles. Drugs were ranked based on their efficacy and safety profiles using the surface under the cumulative ranking curve values, enabling a comprehensive comparative assessment of interventional strategies. The CINeMA (Confidence in Network Meta-Analysis) online tool was utilized to evaluate the confidence level of the NMA results.

**Results:**

This NMA included 22 RCTs and 9,241 patients. All intervention groups demonstrated superior efficacy to the placebo group. Based on efficacy endpoints and subgroup analyses, bimekizumab, secukinumab, and ixekizumab exhibited superior short-term efficacy. Notably, subgroup analyses suggested that tildrakizumab may represent a promising therapeutic option for PsA. Regarding safety and the risk of adverse events, all treatments demonstrated no significant differences compared to placebo, except bimekizumab 160 mg every 4 weeks (Q4W) (OR = 1.37, 95% CI: 1.08–1.74). There were no significant differences in terms of serious adverse events and upper respiratory tract infection. Bimekizumab 160 mg Q4W showed heightened risk of nasopharyngitis (OR = 2.30, 95% CI: 1.26–4.22).

**Conclusions:**

This NMA showed that IL-17, IL-12/23, and IL-23 inhibitors demonstrated remarkable efficacy in attaining ACR20, ACR50, ACR70, and MDA after 12, 16, or 24 weeks of treatment. Among these, IL-17 inhibitors—particularly bimekizumab, secukinumab, and ixekizumab—exhibited notably pronounced therapeutic effects. However, bimekizumab showed a less favorable clinical safety profile compared to other biological agents. In contrast, secukinumab and ixekizumab demonstrated a favorable balance of relatively high efficacy and low risk when considering safety profiles.

**Systematic review registration:**

https://www.crd.york.ac.uk/PROSPERO/view/CRD420251023787, identifier CRD420251023787.

## 1 Introduction

Psoriatic arthritis (PsA) is an enduring inflammatory disorder affecting cutaneous and articular tissues, imposing significant morbidity through synovitis, enthesitis, and dermatological manifestations, thereby compromising patients’ functional capability and life quality (1, 2). Roughly one-third of patients with psoriasis progress to PsA, an estimated annual incidence of PsA of approximately 6 cases per 100,000 individuals, exhibiting no gender predilection (3, 4). A substantial body of literature highlights that patients with PsA face considerable financial stress, as reflected by an average annual medical expenditure of approximately $30,000 per person (5).

The conventional treatment approaches for PsA rely on nonsteroidal anti-inflammatory drugs (NSAIDs) and conventional synthetic disease-modifying antirheumatic drugs (csDMARDs) (6). The recognition of biologics—including tumor necrosis factor (TNF) inhibitors and IL-17 and IL-23 inhibitors—as pivotal in PsA treatment has been driven by advancements in pathogenic mechanisms research, especially for patients unresponsive to DMARD and NSAID regimens (7, 8). Serious infections and exacerbated demyelination episodes have been linked to TNF inhibitor therapy (9). The IL-17 and IL-23 cytokine groups are integral to the operation of innate and adaptive immunity mechanisms, as they participate in multiple cellular signaling pathways, modulate the activation and differentiation of immune cells, and contribute to the balance between proinflammatory and anti-inflammatory responses (10, 11). In psoriasis, IL-23 promotes the proliferation and differentiation of Th17 cells, characterized by the secretion of IL-17, and produces various other proinflammatory cytokines, such as TNF-α and IL-23. A substantial number of Th17 cells then secrete inflammatory cytokines that act on keratinocytes and dendritic cells (DCs), stimulating these cells to produce increased amounts of IL-23, IL-17, and TNF-α (12). These inflammatory cytokines act on keratinocytes and DCs, stimulating these cells to produce increased amounts of IL-23 and other proinflammatory cytokines (13). During this process, activated cells further secrete and recruit inflammatory factors, escalating the inflammatory response and ultimately contributing to the development of chronic inflammatory lesions in psoriasis. Therefore, an array of biologics targeting different cytokine pathways have been explored for PsA management, becoming potential treatment modalities for PsA (14). IL-17 inhibitors (bimekizumab, brodalumab, secukinumab, and ixekizumab), IL-23 inhibitors (guselkumab, tildrakizumab, and risankizumab), and IL-12/23 inhibitors (ustekinumab) have been each proven effective in the treatment of PsA through comprehensive clinical trials (15, 23–43). Previous meta-analyses have studied the efficacy and safety profiles of IL-17 inhibitors in individuals with PsA (16). However, such methodologies yield restricted understanding of the comprehensive treatment prioritization framework. This is primarily due to the fact that treatment efficacy is gauged and reported based solely on a selective portion of pertinent treatment comparisons. Additionally, the lack of direct comparative clinical trials for certain treatment contrasts introduces ambiguity for healthcare professionals and policymakers when making informed decisions.

By integrating a broader spectrum of studies, network meta-analyses (NMAs) enable the indirect comparison of therapies for which direct comparative evidence is lacking. By analyzing multiple studies, NMA boosts statistical power and precision. Not only do network diagrams visualize the evidence network, pinpointing areas for further research, but NMA also ranks interventions based on predefined endpoints. Therefore, this NMA was conducted on all randomized trials, aiming to compare the efficacy and safety of IL-17, IL-12/23, and IL-23 inhibitors in the treatment of PsA.

## 2 Methods

This NMA adhered to the guidelines set forth by the Preferred Reporting Items for Systematic Reviews and Meta-Analyses (PRISMA) extension statement for network meta-analyses (17). The checklist is summarized in **Supplementary Table S1**. This NMA was prospectively registered in PROSPERO with the identifier CRD420251023787 (https://www.crd.york.ac.uk/PROSPERO/view/CRD420251023787).

### 2.1 Search strategy and eligibility criteria

PubMed, Embase, and Web of Science were systematically searched from their respective inceptions up to May 2025. To supplement the search, the ClinicalTrials.gov registry (https://www.clinicaltrials.gov/) was also employed. Two authors (S. Gao and L. Yu) independently screened all the included studies, conducting title and abstract screening for initial literature filtering, followed by full-text retrieval and evaluation based on inclusion criteria. Discrepancies between the authors were adjudicated by a third author (X. Xie). The search strategy is summarized in **Supplementary Table S2**.

Inclusion criteria:

1. Randomized controlled trials (RCTs) enrolling patients with PsA, including biologic-naïve individuals, those withxprior biologic exposure, or subjects with unspecified exposure history.
2. RCTs using IL-17 inhibitors (bimekizumab, brodalumab, secukinumab, and ixekizumab), IL-23 inhibitors (guselkumab, tildrakizumab, and risankizumab), and IL-12/23 inhibitors (ustekinumab) as monotherapy or combination regimens.
3. RCTs evaluating placebo as monotherapy or in combination with PsA standard-of-care treatments.
4. The following outcome measures at least should be reported: American College of Rheumatology response (ACR)20, ACR50, ACR70, minimal disease activity (MDA), and the incidence of adverse events (AEs) and serious adverse events (SAEs).

Exclusion criteria:

1. RCTs that employed different phases from an identical patient cohort.
2. RCTs with ambiguous outcome metrics.
3. Reviews or case reports.

### 2.2 Data extraction and quality assessment

The data derived from RCTs encompassed main author, publication year, NCT number, study design, age, trial phase, dosing regimen for the experimental and control groups, time point, sample size, and outcomes. The outcomes derived from each included study included the number of ACR20, ACR50, ACR70, MDA, AEs, and SAEs. When crossover took place in the studies, pre-crossover data were retrieved for the efficacy outcomes. This NMA incorporated studies with accessible data on week 24. In cases where it was unavailable (or early crossover took place), data from week 12 or 16 were included. Two researchers conducted separate and concurrent screening of the identified records. Any discrepancies were addressed through consensus between the two reviewers or with the aid of a third reviewer. Two researchers independently employed the Cochrane Collaboration’s Trial Bias Risk Assessment Tool to assess every publication incorporated into the analysis and any discrepancies were discussed. Results were processed and reported using Review Manager 5.4 (RevMan 5.4), a software tool for systematic review data analysis.

### 2.3 Data analysis

NMA quantitatively evaluates the comparative therapeutic effects of two or additional interventions and determines the corresponding level of uncertainty (18). Statistical analysis was carried out with the Stata version 14.0 (StataCorp, College Station, TX) (19), comparing the indirect and direct evidence. The network plots were drawn to visualize network geometry and node connectivity. The outcomes of ACR20, ACR50, ACR70, and MDA and the incidence of AEs and SAEs were analyzed. The outcomes were presented as dichotomous data, which were combined as odds ratios (ORs) along with 95% confidence intervals (CIs). An overall test for inconsistency was conducted, and the consistency level was determined based on the *p*-value. The node splitting method was used to test for local inconsistency—discrepancies between direct and indirect effect estimates—within the NMA framework (20, 21). When *p* < 0.05, local inconsistency might be present. Significant inconsistency may compromise the validity of the results. Then, potential sources of discrepancy were investigated. The heterogeneity across study results was evaluated using *I* ^2^ statistics. When *I* ^2^ ≤ 50%, heterogeneity was small. Conversely, *I* ^2^ > 50% indicated substantial heterogeneity. Subgroup analyses were then carried out to detect potential sources of heterogeneity, while sensitivity analyses assessed the robustness and stability of the findings. Heterogeneity was synthesized using estimates of between-study variance (*τ*²) for treatment effects in new studies. The percentage of variability in effect estimates attributed to between-study heterogeneity was summarized via *I* ^2^. In this NMA, the surface under the cumulative ranking curve (SUCRA) was employed to rank the intervention hierarchy. A SUCRA value that approaches 1 implies a greater likelihood of a particular treatment being one of the top-ranked interventions or the most optimal choice in general.

### 2.4 Certainty assessment

CINeMA (Confidence in Network Meta-Analysis) was employed to assess each comparison credibility (22). This tool has six aspects as follows: within-study bias, indirectness, reporting bias, imprecision, inconsistency, and heterogeneity. Each domain was categorized as no concern (no downgrade), some concern (one-level downgrade), or major concern (two-level downgrade) according to the severity of bias. We aggregated these domain-specific judgments to derive an overall confidence rating, which ranged from very low, low, moderate, to high and listed all the downgrading reasons for each comparison.

## 3 Results

### 3.1 Study selection and characteristics

**Figure 1:**
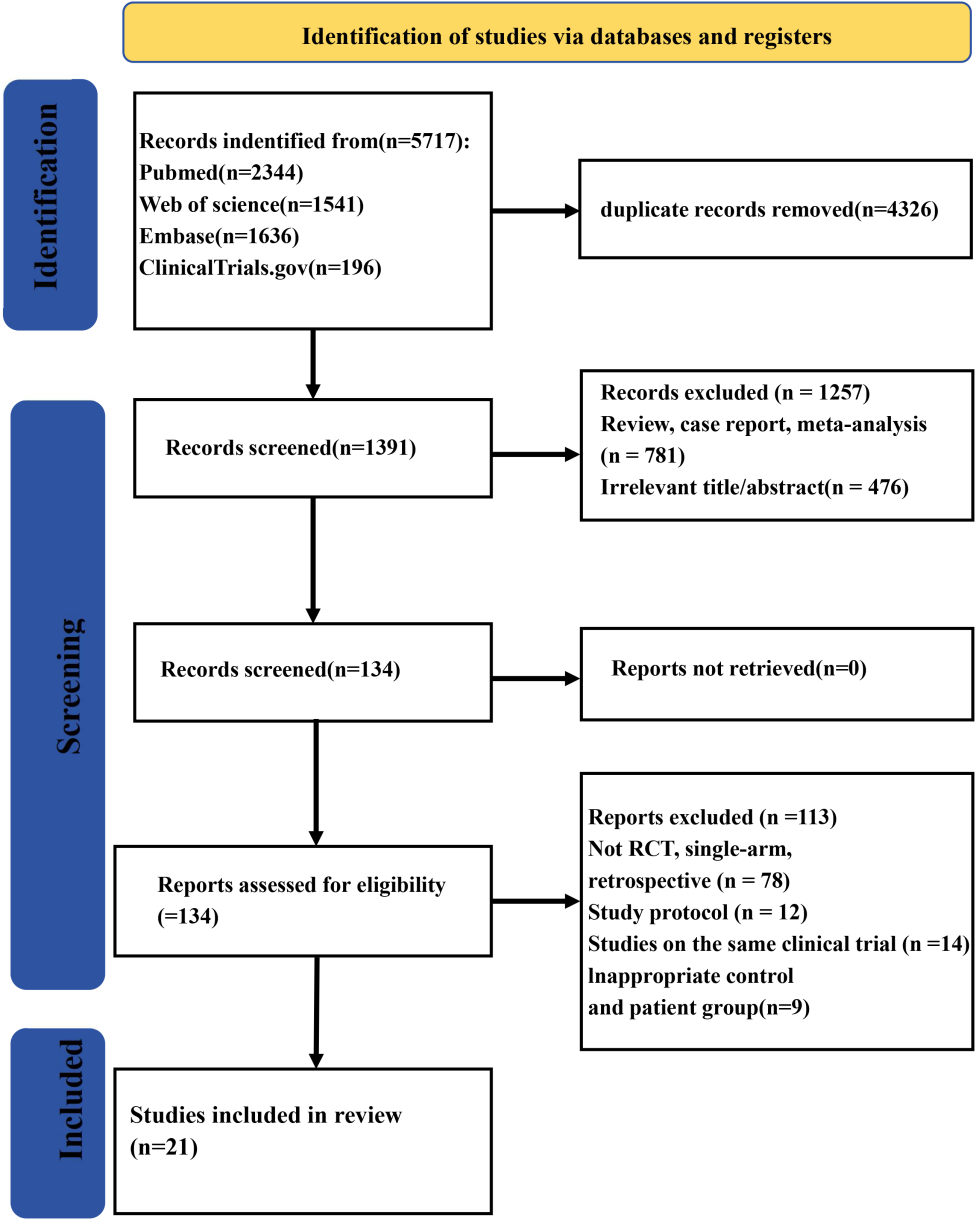
Flow diagram of the study screening process.

A total of 5,717 records were collected from multiple databases. Following the screening of abstracts to eliminate 4,326 duplicate entries, 1,391 studies were retained for full-text review. Among these, one was excluded owing to being meta-analyses/review (*n* = 781) or irrelevance (*n* = 476). Of the remaining 134 reports, 113 were eliminated during the eligibility evaluation process due to various reasons such as 78 having non-RCT, single-arm, or retrospective study designs; 12 being study protocols; 14 being the same clinical trials; or 9 having inadequate patient and control groups. Finally, 21 studies (23–43) were qualified for eligibility criteria. The study screening process is shown in **Figure 1**.

This NMA involved 22 RCTs and 9,241 patients. Excluding four phase II trials, the remaining ones were all phase III trials. Outcomes were assessed in 3 trials at week 12, 5 trials at week 16, and 14 trials at week 24. The characteristics of the included studies are detailed in **Table 1**.

**Table 1:** Characteristics of the included studies.

Author, year	Trial number	Study design	Age	Interventions	Number of patients	Time point (weeks)	Outcomes included in NMA
Ritchlin et al., 2020 (23)	NCT02969525	Phase IIb double-blind RCT	48.0 (11.7) 49.0 (12.1)	BKZ 160 mg Q4W Placebo	*n* = 41 *n* = 42	12	ACR20/50/70, MDA, PASI75/90/100, AEs/SAEs
Merola et al., 2023 (24)	NCT03896581	Phase III double-blind RCT	50.1 (12.4) 51.3 (12.9)	BKZ 160 mg Q4W Placebo	*n* = 267 *n* = 133	16	ACR20/50/70, MDA, PASI75/90/100, AEs/SAEs
McInnes et al., 2023 (25)	NCT03895203	Phase III double-blind RCT	48.5 (12.6) 48.7 (11.7)	BKZ 160 mg Q4W Placebo	*n* = 431 *n* = 281	16	ACR20/50/70, MDA, PASI75/90/100, AEs/SAEs
Mease et al., 2021^a^ (26)	NCT02024646	Phase III double-blind RCT	47.0 (12.6) 47.4 (12.8) 48.3 (13.0)	BRO 210 mg Q2W BRO 140 mg Q2W Placebo	*n* = 163 *n* = 160 *n* = 161	16	ACR20/50/70 PASI75/90/100
Mease et al., 2021^b^ (26)	NCT02029495	Phase III double-blind RCT	49.1 (12.2) 49.9 (12.8) 48.1 (11.8)	BRO 210 mg Q2W BRO 140 mg Q2W Placebo	*n* = 158 *n* = 159 *n* = 161	16	ACR20/50/70 PASI75/90/100
Mease et al., 2017 (27)	NCT01695239	Phase III double-blind RCT	49.8 (12.6) 49.1 (10.1) 50.6 (12.3)	IXE 80 mg Q2W IXE 80 mg Q4W Placebo	*n* = 103 *n* = 107 *n* = 106	24	ACR20/50/70 PASI75/90/100, AEs/SAEs
Nash et al., 2017 (28)	NCT02349295	Phase III double-blind RCT	51.7 (11.9) 52.6 (13.6) 51.5 (10.4)	IXE 80 mg Q2W IXE 80 mg Q4W Placebo	*n* = 123 *n* = 122 *n* = 118	24	ACR20/50/70, MDA PASI75/90/100 AEs/SAEs
Nash et al., 2018 (29)	NCT01989468	Phase III double-blind RCT	49.3 (12.9) 50.1 (11.7) 50.1 (12.6)	SEC 300 mg Q4W SEC 150 mg Q4W Placebo	*n* = 137 *n* = 139 *n* = 138	24	ACR20/50/70, MDA,PASI75/90/100, AEs/SAEs
Kivitz et al., 2019 (30)	NCT02294227	Phase III double-blind RCT	48.3 (12.2) 48.5 (12.2)	SEC 150 mg Q4W Placebo	*n* = 114 *n* = 114	16	ACR20/50/70, MDA, PASI75/90/100, AEs/SAEs
McInnes et al., 2015 (31)	NCT01752634	Phase III double-blind RCT	46.9 (12.6) 46.5 (11.7) 49.9 (12.5)	SEC 300 mg Q4W SEC 150 mg Q4W Placebo	*n* = 98 *n* = 100 *n* = 100	24	ACR20/50/70 PASI75/90 AEs/SAEs
Mease et al., 2018 (32)	NCT02404350	Phase III double-blind RCT	48.9 (12.8) 48.4 (12.9) 49.0 (12.1)	SEC 300 mg Q4W SEC 150 mg Q4W Placebo	*n* = 220 *n* = 222 *n* = 332	24	ACR20/50/70 MDA PASI75/90
Baraliakos et al., 2021 (33)	NCT02721966	Phase IIIb double-blind RCT	46.2 (12.3) 46.9 (11.5) 46.6 (11.5)	SEC 300 mg Q4W SEC 150 mg Q4W Placebo	*n* = 167 *n* = 165 *n* = 166	12	ACR20 AEs/SAEs
Mease PJ et al., 2021 (34)	NCT02980692	Phase IIb double-blind RCT	50.1 ± 13.3 48.1 ± 13.3	TLD 200 mg Q4W Placebo	*n* = 78 *n* = 79	24	ACR20/50/70, MDA, PASI75/90, AEs/SAEs
Kristensen et al., 2022 (35)	NCT03675308	Phase III double-blind RCT	52 (20–85) 52 (22–79)	RIS 150 mg Placebo	*n* = 483 *n* = 481	24	ACR20/50/70, MDA, PASI 90, AEs/SAEs
Ostor et al., 2022 (36)	NCT03671148	Phase III double-blind RCT	53 (23–84) 52 (24– 83)	RIS 150 mg Placebo	*n* = 224 *n* = 219	24	ACR20/50/70, MDA, PASI 90, AEs/SAEs
Mease et al., 2020 (37)	NCT03158285	Phase IIIb double-blind RCT	45.9(11.5) 44.9 (11.9) 46.3 (11.7)	GUS 100 mg Q4W GUS 100 mg Q8W Placebo	*n* = 246 *n* = 248 *n* = 247	24	ACR20/50/70, MDA, PASI75/90/100, AEs/SAEs
Deodhar et al., 2020 (38)	NCT03162796	Phase III double-blind RCT	49 (12) 49 (12)	GUS 100 mg Q4W GUS 100 mg Q8W Placebo	*n* = 128 *n* = 127 *n* = 126	24	ACR20/50/70, MDA, PASI75/90/100, AEs/SAEs
Coates et al., 2022 (39)	NCT03796858	Phase III double-blind RCT	49 (12) 49 (12)	GUS 100 mg Q8W Placebo	*n* = 189 *n* = 96	24	ACR20/50/70, MDA, PASI75/90/100, AEs/SAEs
Deodhar et al., 2018 (40)	NCT02319759	Phase IIb double-blind RCT	47.4 (12.8) 44.2 (12.4)	GUS 100 mg Q8W Placebo	*n* = 100 *n* = 49	24	ACR20/50/70, MDA, PASI75/90/100, AEs/SAEs
Ritchlin et al., 2014 (41)	NCT01077362	Phase III double-blind RCT	49.0(40.0- 56.0) 48.0(38.5 -56.0) 48.0(41.0- 57.0)	UST 45 mg Q12W UST 90 mg Q12W Placebo	*n* = 103 *n* = 105 *n* = 104	24	ACR20/50/70 PASI75/90/100 AEs/SAEs
McInnes et al., 2013 (42)	NCT01009086	Phase III double-blind RCT	48.0 (39.0–55.0) 47.0 (38.5–54.0) 48.0 (39.0–57.0)	UST 45 mg Q12W UST 90 mg Q12W Placebo	*n* = 205 *n* = 204 *n* = 206	24	ACR20/50/70 AEs/SAEs
Gottlieb et al., 2009 (43)	NCT00267956	Phase II double-blind RCT	50.0 (42.0–60.5) 47.5 (40.0–55.0)	UST 90 mg Q12W Placebo	*n* = 76 *n* = 70	12	ACR20/50/70 PASI75/90, AEs/SAEs

ACR, American College of Rheumatology response; MDA, minimal disease activity; AEs, adverse events; SAEs, serious adverse events; PBO, Placebo; BKZ, bimekizumab; BRO, brodalumab; IXE, ixekizumab; SEC, secukinumab; TLD, tildrakizumab; RIS, risankizumab; GUS, guselkumab; UST, ustekinumab. One 2021 publication reported data for both AMVISION-1 and AMVISION-2, a: AMVISION-2; b:AMVISION-1.

### 3.2 Quality assessment and risk of bias

**Figure 2:**
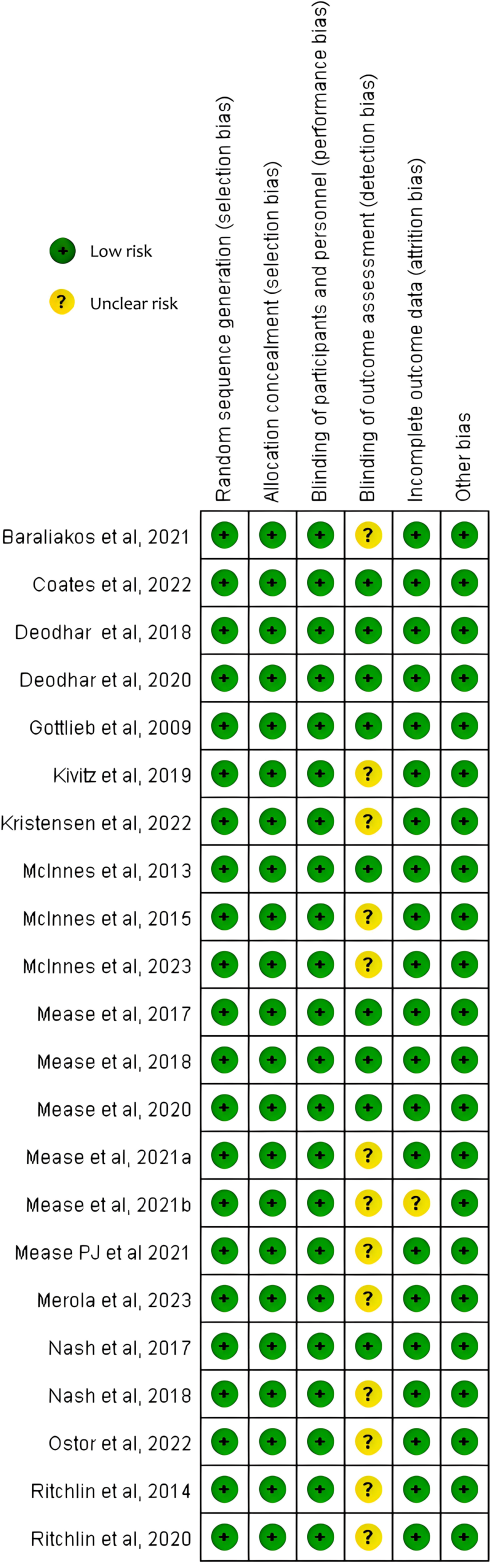
Risk of bias of the included studies.

The Cochrane Collaboration’s Trial Bias Risk Assessment Tool was used to appraise study quality. Overall, all the included studies were deemed to have a low risk of bias and no studies have moderate or high risk. The risk of bias is summarized in **Figure 2** and **Supplementary Table S3**.

### 3.3 Inconsistency analysis

Inconsistency denotes the disparity between direct and indirect evidence, and this phenomenon has the potential to impact the validity of NMA. To derive inconsistency between direct and indirect comparison, we employed the node-splitting approach. There is no global and local inconsistency observed (**Supplementary Table S4** and **Supplementary Figure S1**).

### 3.4 Network meta-analysis

#### 3.4.1 Network plot

**Figure 3:**
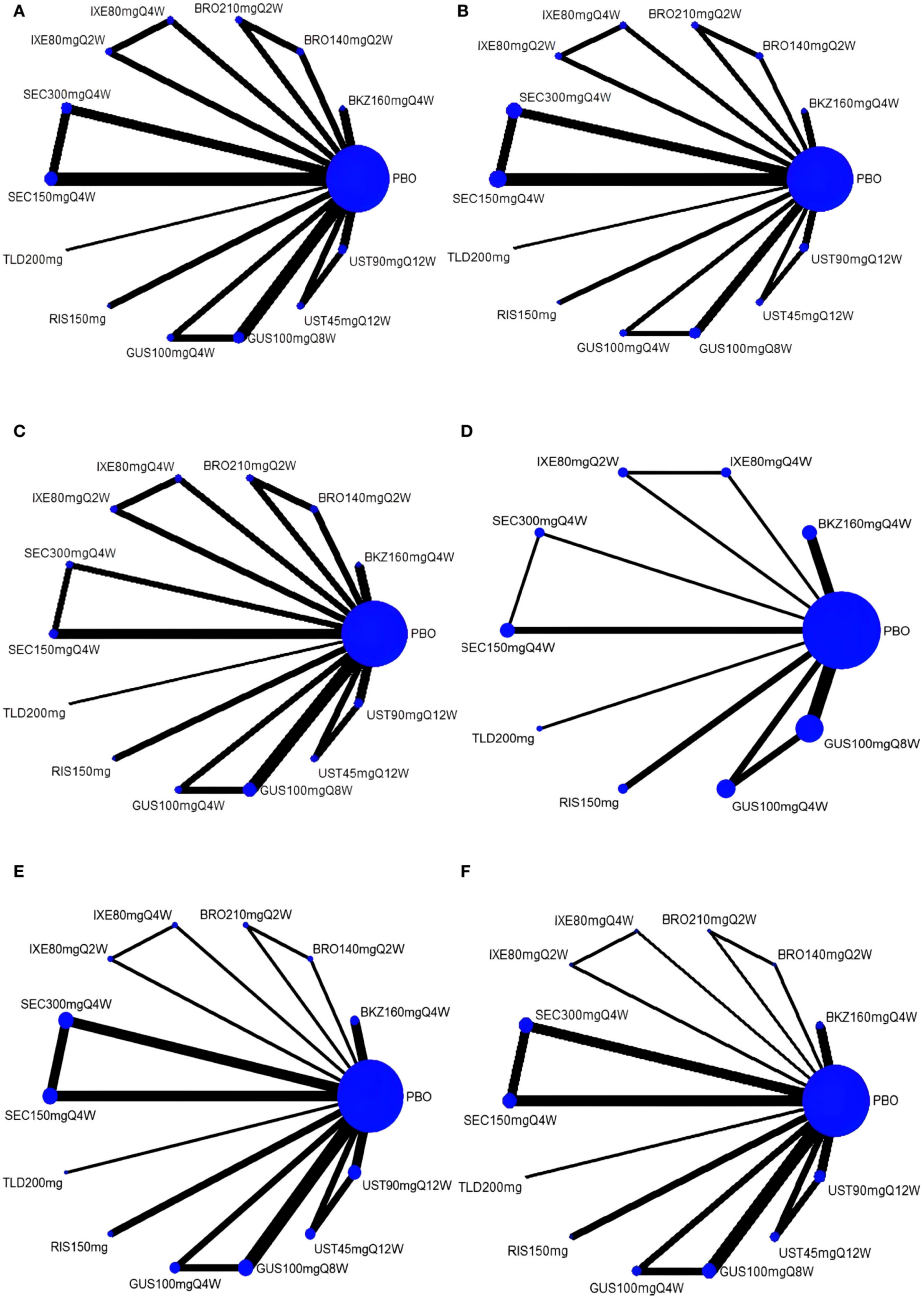
Network plots for four outcomes. **(A)** ACR20; **(B)** ACR50; **(C)** ACR70; **(D)** MDA; **(E)** AEs; **(F)** SAEs. The magnitudes of the nodes are weighted based on the sample volume of interventions, and the thicknesses of the lines are weighted according to the count of included studies. ACR, American College of Rheumatology response; MDA, minimal disease activity; AEs, adverse events; SAEs, serious adverse events; PBO, placebo; BKZ, bimekizumab; BRO, brodalumab; IXE, ixekizumab; SEC, secukinumab; TLD, tildrakizumab; RIS, risankizumab; GUS, guselkumab; UST, ustekinumab.

Six networks were built to show the primary outcomes. Five network diagrams incorporated 14 interventions and one network diagram included 10 interventions. The network plots are presented in **Figure 3**. Among all interventions, the 150-mg dosage of secukinumab had the highest frequency in terms of both the number of studies and participants.

#### 3.4.2 Efficacy analysis

##### 3.4.2.1 ACR 20

**Figure 4:**
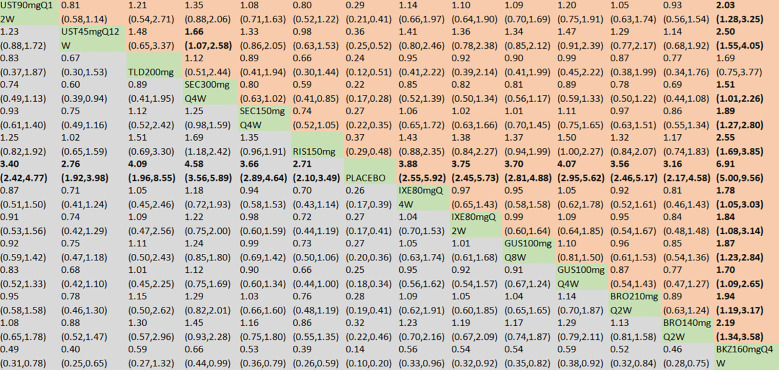
ACR20 was presented using OR with 95% CIs of all interventions. The central block of different interventions splits the graph into upper and lower triangular sections. For the lower triangle, the efficacy estimate represents the ratio of the column-defined treatment to the row-defined treatment. When OR > 1 in the lower triangle, it indicates superiority of the column-defined treatment, whereas an OR < 1 favors the row-defined treatment. The upper triangle mirrors the lower triangle symmetrically. Statistically significant results are highlighted in bold formatting. BKZ, bimekizumab; BRO, brodalumab; IXE, ixekizumab; SEC, secukinumab; TLD, tildrakizumab; RIS, risankizumab; GUS, guselkumab; UST, ustekinumab.

With regard to the ACR20 response, a total of 22 RCTs and 14 interventions were included (**Figure 3A**). In the consistency model, the heterogeneity was small with an *I* ^2^ of 9% and a *τ* ^2^ of 1.15. In the direct comparisons, all the treatments were superior to placebo (**Supplementary Figure S2A**). In the mixed comparisons, bimekizumab 160 mg Q4W demonstrated greater efficacy than brodalumab 140 mg every 2 weeks (Q2W) (OR = 2.19, 95% CI: 1.34–3.58), brodalumab 210 mg Q2W (OR = 1.94, 95% CI: 1.19–3.17), guselkumab 100 mg Q4W (OR = 1.70, 95% CI: 1.09–2.65), guselkumab 100 mg every 8 weeks (Q8W) (OR = 1.87, 95% CI: 1.23–2.84), ixekizumab 80 mg Q2W (OR = 1.84, 95% CI: 1.08–3.14), ixekizumab 80 mg Q4W (OR = 1.78, 95% CI: 1.05–3.03), risankizumab 150 mg (OR = 2.55, 95% CI: 1.69–3.85), secukinumab 150 mg Q4W (OR = 1.89, 95% CI: 1.27–2.80), secukinumab 300 mg Q4W (OR = 1.51, 95% CI: 1.01–2.26), ustekinumab 45 mg every 12 weeks (Q12W) (OR = 2.50, 95% CI: 1.55–4.05), and ustekinumab 90 mg Q12W (OR = 2.03, 95% CI: 1.28–3.25). Secukinumab 300 mg Q4W showed superior efficacy to ustekinumab 45 mg Q12W (OR = 1.66, 95% CI: 1.07–2.58) (**Figure 4**). No significant differences were observed between secukinumab 300 mg Q4W and secukinumab 150 mg Q4W (OR = 1.25, 95% CI: 0.98–1.59), between ixekizumab 80 mg Q4W and ixekizumab 80 mg Q2W (OR = 1.04, 95% CI: 0.70–1.53), between ustekinumab 90 mg Q12W and ustekinumab 45 mg Q12W (OR = 1.23, 95% CI: 0.88–1.72), between guselkumab 100 mg Q8W and guselkumab 100 mg Q4W (OR = 0.91, 95% CI: 0.67–1.24), or between brodalumab 210 mg Q2W and brodalumab 140 mg Q2W (OR = 1.13, 95% CI: 0.81–1.58). Based on SUCRA values, ranking from high to low was as follows: bimekizumab 160 mg Q4W (SUCRA, 98.6%), secukinumab 300 mg Q4W (SUCRA, 80.3%), and guselkumab 100 mg Q8W (SUCRA, 66.3%). The summarized SUCRA of ACR20 is shown in **Table 2**.

**Table 2:** SUCRAs of all interventions according to ACR20, ACR50, ACR70, MDA, AEs, and SAEs.

	Efficacy				Safety	
Interventions	ACR20 (%)	ACR50 (%)	ACR70 (%)	MDA (%)	AEs (%)	SAE (%)
Placebo	0.0	0	0.1	0.1	56.9	41.3
Bimekizumab 160 mg Q4W	98.6	93.6	81.7	76.2	13.0	20.5
Brodalumab 140 mg Q2W	34.9	49.5	36.2	NA	73.4	66.5
Brodalumab 210 mg Q2W	49.7	61.7	44.8	NA	55.3	32.1
Ixekizumab 80 mg Q4W	59.8	67.5	80.5	89.6	36.3	61.6
Ixekizumab 80 mg Q2W	55.4	74.1	77.3	79.5	13.0	16.6
Secukinumab 300 mg Q4W	80.3	84.4	73.6	65.4	77.5	50.0
Secukinumab 150 mg Q4W	52.7	59.2	71.7	51.7	82.4	82.2
Tildrakizumab 200 mg Q4W	63.0	33.8	37.1	29.9	76.7	45.8
Risankizumab 150 mg	18.8	36.2	24.7	21.7	46.0	67.4
Guselkumab 100 mg Q4W	66.3	40.1	46.3	39.7	45.6	41.5
Guselkumab 100 mg Q8W	54.4	28.9	47.5	46.2	55.5	61.9
Ustekinumab 45 mg Q12W	21.8	26.0	32.7	NA	25.8	47.7
Ustekinumab 90 mg Q12W	44.3	44.9	45.7	NA	42.5	65.1

ACR, American College of Rheumatology response; MDA, minimal disease activity; AEs, adverse events; SAEs, serious adverse events.

##### 3.4.2.2 ACR50

**Figure 5:**
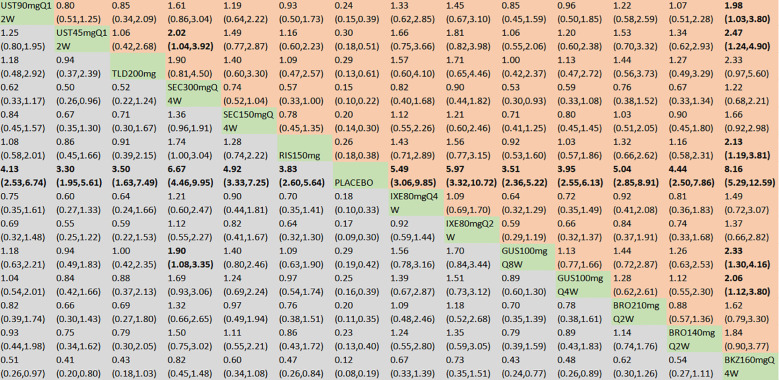
ACR50 was presented using OR with 95% CIs of all interventions. The central block of different interventions splits the graph into upper and lower triangular sections. For the lower triangle, the efficacy estimate represents the ratio of the column-defined treatment to the row-defined treatment. When OR > 1 in the lower triangle, it indicates superiority of the column-defined treatment, whereas an OR < 1 favors the row-defined treatment. The upper triangle mirrors the lower triangle symmetrically. Statistically significant results are highlighted in bold formatting. BKZ, bimekizumab; BRO, brodalumab; IXE, ixekizumab; SEC, secukinumab; TLD, tildrakizumab; RIS, risankizumab; GUS, guselkumab; UST, ustekinumab.

With regard to the ACR50 response, a total of 21 RCTs and 14 interventions were included (**Figure 3B**). In the consistency model, the heterogeneity was small with an *I* ^2^ of 1% and a *τ* ^2^ of 0.43. In the direct comparisons, all the treatments were superior to placebo (**Supplementary Figure S2B**). In the mixed comparisons, bimekizumab 160 mg Q4W demonstrated superior efficacy to guselkumab 100 mg Q4W (OR = 2.06, 95% CI: 1.12–3.80), guselkumab 100 mg Q8W (OR = 2.33, 95% CI: 1.30–4.16), risankizumab 150 mg (OR = 2.13, 95% CI: 1.19–3.81), ustekinumab 45 mg Q12W (OR = 2.47, 95% CI: 1.24–4.90), and ustekinumab 90 mg Q12W (OR = 1.98, 95% CI: 1.03–3.80). Secukinumab 300 mg Q4W demonstrated statistically significant superiority to guselkumab 100 mg Q8W (OR = 1.90, 95% CI: 1.08–3.35) and ustekinumab 45 mg Q12W (OR = 2.02, 95% CI: 1.04–3.92) (**Figure 5**). No significant differences were observed between secukinumab 300 mg Q4W and secukinumab 150 mg Q4W (OR = 1.36, 95% CI: 0.96–1.91), between ixekizumab 80 mg Q4W and ixekizumab 80 mg Q2W (OR = 0.92, 95% CI: 0.59–1.44), between ustekinumab 90 mg Q12W and ustekinumab 45 mg Q12W (OR = 1.25, 95% CI: 0.80–1.95), between guselkumab 100 mg Q8W and guselkumab 100 mg Q4W (OR = 0.89, 95% CI: 0.60–1.30), or between brodalumab 210 mg Q2W and brodalumab 140 mg Q2W (OR = 1.14, 95% CI: 0.74–1.76). Based on SUCRA values, ranking from high to low was as follows: bimekizumab 160 mg Q4W (SUCRA, 93.6%), secukinumab 300 mg Q4W (SUCRA, 84.4%), and ixekizumab 80 mg Q2W (SUCRA, 74.1%). The summarized SUCRA of ACR50 is shown in **Table 2**.

##### 3.4.2.3 ACR70

**Figure 6:**
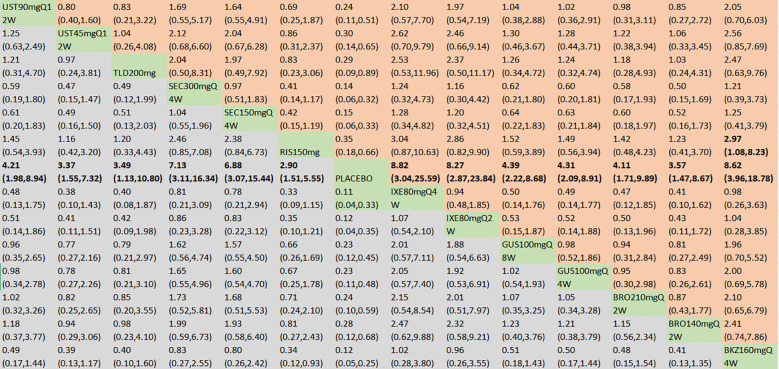
ACR70 was presented using OR with 95% CIs of all interventions. The central block of different interventions splits the graph into upper and lower triangular sections. For the lower triangle, the efficacy estimate represents the ratio of the column-defined treatment to the row-defined treatment. When OR > 1 in the lower triangle, it indicates superiority of the column-defined treatment, whereas an OR < 1 favors the row-defined treatment. The upper triangle mirrors the lower triangle symmetrically. Statistically significant results are highlighted in bold formatting. BKZ, bimekizumab; BRO, brodalumab; IXE, ixekizumab; SEC, secukinumab; TLD, tildrakizumab; RIS, risankizumab; GUS, guselkumab; UST, ustekinumab.

With regard to the ACR70 response, a total of 21 RCTs and 14 intervention nodes were included (**Figure 3C**). In the consistency model, the heterogeneity was small with an *I* ^2^ of 3% and a *τ* ^2^ of 0.14. In the direct comparisons, all the treatments demonstrated significant differences compared to placebo (**Supplementary Figure S2C**). In the mixed comparisons, bimekizumab 160 mg Q4W demonstrated superior efficacy to risankizumab 150 mg (OR = 2.97, 95% CI: 1.08–8.23) (**Figure 6**). No significant differences were observed between secukinumab 300 mg Q4W and secukinumab 150 mg Q4W (OR = 1.04, 95% CI: 0.55–1.96), between ixekizumab 80 mg Q4W and ixekizumab 80 mg Q2W (OR = 1.07, 95% CI: 0.54–2.10), between ustekinumab 90 mg Q12W and ustekinumab 45 mg Q12W (OR = 1.25, 95% CI: 0.63–2.49), between guselkumab 100 mg Q8W and guselkumab 100 mg Q4W (OR = 1.02, 95% CI: 0.54–1.93), or between brodalumab 210 mg Q2W and brodalumab 140 mg Q2W (OR = 1.15, 95% CI: 0.56–2.34). Based on SUCRA values, ranking from high to low was as follows: bimekizumab 160 mg Q4W (SUCRA, 81.7%), ixekizumab 80 mg Q4W (SUCRA, 80.5%), and ixekizumab 80 mg Q2W (SUCRA, 77.3%). The summarized SUCRA of ACR70 is shown in **Table 2**.

##### 3.4.2.4 MDA

**Figure 7:**
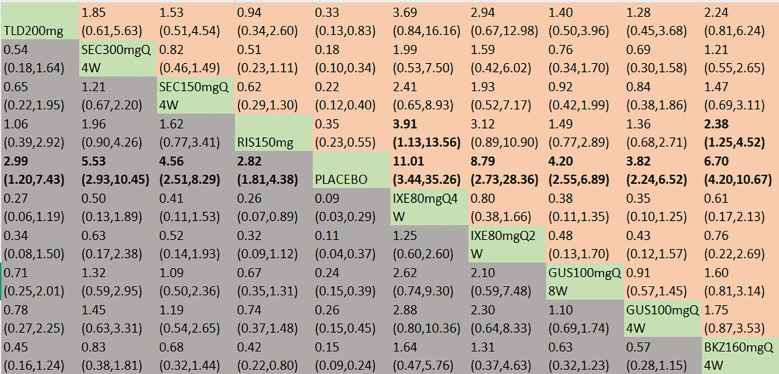
MDA was presented using OR with 95% CIs of all interventions. The central block of different interventions splits the graph into upper and lower triangular sections. For the lower triangle, the efficacy estimate represents the ratio of the column-defined treatment to the row-defined treatment. When OR > 1 in the lower triangle, it indicates superiority of the column-defined treatment, whereas an OR < 1 favors the row-defined treatment. The upper triangle mirrors the lower triangle symmetrically. Statistically significant results are highlighted in bold formatting. BKZ, bimekizumab; BRO, brodalumab; IXE, ixekizumab; SEC, secukinumab; TLD, tildrakizumab; RIS, risankizumab; GUS, guselkumab; UST, ustekinumab.

With regard to the MDA response, a total of 13 RCTs and 10 interventions were included (**Figure 3D**). In the consistency model, the heterogeneity was small with an *I* ^2^ of 4% and a *τ* ^2^of 0.11. In the direct comparisons, all the treatments were significantly effective compared to placebo (**Supplementary Figure S2D**). In the mixed comparisons, bimekizumab 160 mg Q4W demonstrated superior efficacy to risankizumab 150 mg (OR = 2.38, 95% CI:1.25–4.52). Ixekizumab 80 mg Q4W demonstrated superior efficacy to risankizumab 150 mg (OR = 3.91, 95% CI: 1.13–13.56) (**Figure 7**). No significant differences were observed between secukinumab 300 mg Q4W and secukinumab 150 mg Q4W (OR = 1.21, 95% CI: 0.67–2.20), between ixekizumab 80 mg Q4W and ixekizumab 80 mg Q2W (OR = 1.25, 95% CI: 0.60–2.60), or between guselkumab 100 mg Q8W and guselkumab 100 mg Q4W (OR = 1.10, 95% CI: 0.69–1.74). Based on SUCRA values, ranking from high to low was as follows: ixekizumab 80 mg Q4W (SUCRA, 89.6%), ixekizumab 80 mg Q2W (SUCRA, 79.5%), and bimekizumab 160 mg Q4W (SUCRA, 76.2%). The summarized SUCRA of MDA is shown in **Table 2**.

##### 3.4.2.5 Subgroup analysis according to prior anti-TNF exposure

Eleven clinical trials evaluated the impact of prior anti-TNF agent exposure on ACR20 response outcomes in patients with PsA. In terms of anti-TNF exposure patients, based on SUCRA values, ranking from high to low was as follows: bimekizumab 160 mg Q4W (SUCRA, 95.3%), guselkumab 100 mg Q4W (SUCRA, 73.1%), and guselkumab 100 mg Q8W (SUCRA, 72.1%). In terms of anti-TNF-naïve patients, based on SUCRA values, ranking from high to low was as follows: bimekizumab 160 mg Q4W (SUCRA, 89.9%), secukinumab 300 mg Q4W (SUCRA, 79.5%), and tildrakizumab 200 mg Q4W (SUCRA, 71.2%). Eight studies assessed the influence of prior anti-TNF agent exposure on ACR50 response outcomes in patients with PsA. In terms of anti-TNF exposure patients, based on SUCRA values, ranking from high to low was as follows: bimekizumab 160 mg Q4W (SUCRA, 70.4%), ixekizumab 80 mg Q4W (SUCRA, 69.4%), and tildrakizumab 200 mg Q4W (SUCRA, 66.9%). In terms of anti-TNF-naïve patients, bimekizumab 160 mg Q4W ranked the highest in SUCRA value (SUCRA, 74.0%). For ACR70, seven clinical trials examined the effects of prior anti-TNF agent exposure. Ixekizumab 80 mg Q4W showed the highest SUCRA value in anti-TNF exposure patients (SUCRA, 80.9%), followed by bimekizumab 160 mg Q4W (SUCRA, 76.6%). In the anti-TNF-naïve subgroup, based on SUCRA values, ranking from high to low was as follows: ixekizumab 80 mg Q2W (SUCRA, 84.0%), secukinumab 150 mg Q4W (SUCRA, 83.6%), and bimekizumab 160 mg Q4W (SUCRA, 76.8%). Summary data on SUCRA values stratified by prior anti-TNF exposure are presented in **Table 3**.

**Table 3:** SUCRAs of all interventions according to prior anti-TNF exposure.

Interventions	ACR20		ACR50		ACR70	
	Anti-TNF exposure (%)	Anti-TNF-naïve (%)	Anti-TNF exposure (%)	Anti-TNF-naïve (%)	Anti-TNF exposure (%)	Anti-TNF-naïve (%)
Placebo	0.7	0	0.4	3.8	6.7	0.1
Bimekizumab 160 mg Q4W	95.3	89.9	70.4	74.0	76.6	76.8
Ixekizumab 80 mg Q4W	57.1	40.2	69.4	52.9	80.9	54.7
Ixekizumab 80 mg Q2W	40.8	58.6	63.2	62.5	68.3	84.0
Secukinumab 300 mg Q4W	63.0	79.5	45.4	58.3	28.2	78.8
Secukinumab 150 mg Q4W	42.0	53.5	20.4	32.3	45.3	83.6
Tildrakizumab 200 mg Q4W	30.4	71.2	66.9	46.0	48.8	27.2
Risankizumab 150 mg	56.9	25.8	NA	54.9	NA	33.3
Guselkumab 100 mg Q4W	73.1	62.4	65.2	55.8	63.4	32.1
Guselkumab 100 mg Q8W	72.1	50.4	48.7	51.4	31.7	50.9
Ustekinumab 45 mg Q12W	37.1	20.3	NA	50.5	NA	32.5
Ustekinumab 90 mg Q12W	31.6	48.1	NA	57.5	NA	46.0

#### 3.4.3 Safety analysis

**Figure 8:**
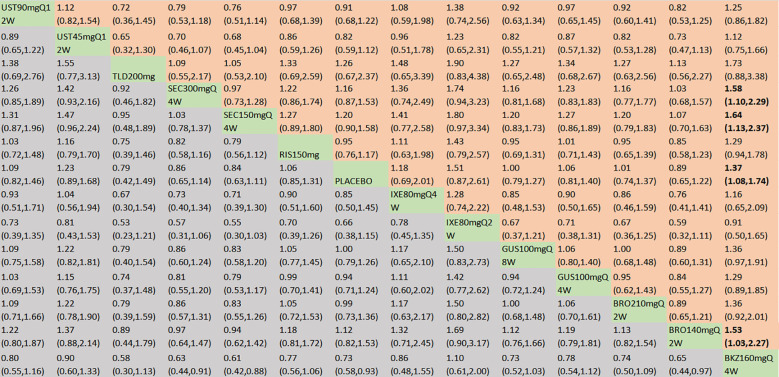
AEs were presented using OR with 95% CIs of all interventions. The central block of different interventions splits the graph into upper and lower triangular sections. For the lower triangle, the efficacy estimate represents the ratio of the column-defined treatment to the row-defined treatment. When OR > 1 in the lower triangle, it indicates superiority of the column-defined treatment, whereas an OR < 1 favors the row-defined treatment. The upper triangle mirrors the lower triangle symmetrically. Statistically significant results are highlighted in bold formatting. BKZ, bimekizumab; BRO, brodalumab; IXE, ixekizumab; SEC, secukinumab; TLD, tildrakizumab; RIS, risankizumab; GUS, guselkumab; UST, ustekinumab.

**Figure 9:**
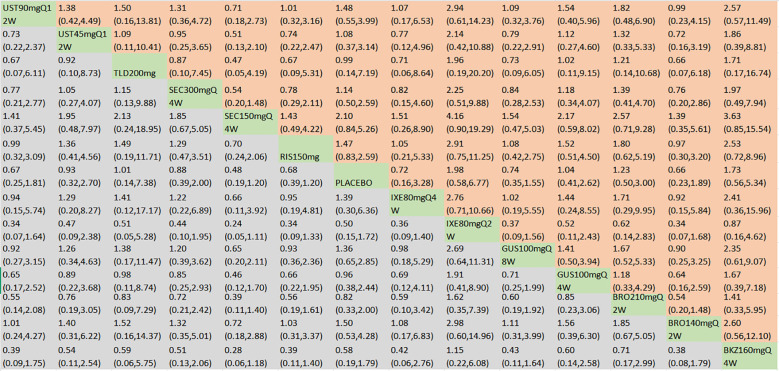
SAEs were presented using OR with 95% CIs of all interventions. The central block of different interventions splits the graph into upper and lower triangular sections. For the lower triangle, the efficacy estimate represents the ratio of the column-defined treatment to the row-defined treatment. When OR > 1 in the lower triangle, it indicates superiority of the column-defined treatment, whereas an OR < 1 favors the row-defined treatment. The upper triangle mirrors the lower triangle symmetrically. Statistically significant results are highlighted in bold formatting. BKZ, bimekizumab; BRO, brodalumab; IXE, ixekizumab; SEC, secukinumab; TLD, tildrakizumab; RIS, risankizumab; GUS, guselkumab; UST, ustekinumab.

In terms of the incidence of AEs, we included 18 RCTs and 14 interventions nodes (**Figure 3E**). In the consistency model, the heterogeneity was small with an *I* ^2^ of 8% and a *τ* ^2^ of 0.05. In the direct comparisons, all the treatments demonstrated no significant differences compared to placebo, except bimekizumab 160 mg Q4W (OR = 1.37, 95% CI: 1.08–1.74) (**Supplementary Figure S2E**). In the mixed comparisons, bimekizumab 160 mg Q4W demonstrated a higher rate than brodalumab 140 mg Q2W (OR = 1.53, 95% CI: 1.03–2.27), secukinumab 150 mg Q4W (OR = 1.64, 95% CI: 1.13–2.37), and secukinumab 300 mg Q4W (OR = 1.58, 95% CI: 1.10–2.29) (**Figure 8**). In terms of the incidence of SAEs, 18 RCTs and 14 interventions were included (**Figure 3F**). In the consistency model, the heterogeneity was small with an *I* ^2^ of 8% and a *τ* ^2^ of 1.58. There is no significant differences in mixed and direct comparisons (**Supplementary Figure S2F**; **Figure 9**). Bimekizumab 160 mg Q4W showed heightened risks of nasopharyngitis (OR = 2.30, 95% CI: 1.26–4.22) (**Supplementary Figure S2G**). There was no significant difference in terms of upper respiratory tract infection (**Supplementary Figure S2H**).

### 3.5 Certainty of evidence

The certainty of evidence for six outcomes is shown in **Supplementary Table S5**. Across the analysis of all six outcomes, it was found that the confidence for 61% of placebo comparisons was classified as either high or moderate (**Supplementary Figure S3**). The confidence in the evidence of 79% was rated as low during pairwise drug comparisons, largely due to factors including imprecision, heterogeneity, or incoherence.

### 3.6 Publication bias

Funnel plots of ACR20, ACR50, ACR70, and AEs are shown in **Supplementary Figure S4**. The funnel plot shows no evidence of potential bias sources for ACR50 and AEs. Findings for ACR20 and ACR70 lacked absolute symmetry, indicating potential publication bias.

### 3.7 Sensitivity analysis

Since the analysis incorporates outcomes assessed at varying time points (12, 16, and 24 weeks), we performed sensitivity analyses on ACR20 by sequentially excluding each time point to gauge the stability of our results. Efficacy outcomes aligned with those from the primary analysis, and comprehensive SUCRA data are presented in **Supplementary Figures S5**-**S7**.

## 4 Discussion

PsA represents a long-standing, systemic inflammatory condition that imposes a substantial disease burden on affected individuals (44). The clinical efficacy of therapeutic molecules that selectively target IL-17 and IL-23 has been notably proven in psoriasis and PsA, and its associated interleukin pathway has emerged as a novel therapeutic target (45). Therefore, interleukin inhibitors have emerged as a novel clinical treatment modality for PsA. Nevertheless, no definitive conclusion has been established regarding which interleukin inhibitors demonstrate optimal therapeutic efficacy in the treatment of PsA. This study firstly aimed to employ NMA to compare the efficacy and safety of IL-17, IL-12/23, and IL-23 Inhibitors in the treatment of PsA, with the objective of offering clinical guidance for these inhibitors’ utilization and informing the design of subsequent related research.

This NMA of IL-17, IL-12/23, and IL-23 inhibitors in PsA treatment aggregated data from 22 clinical trials, encompassing a total of 9,241 patients with PsA. Except for four phase II trials (23, 34, 40, 43), the remaining ones were all phase III trials. The evidence quality predominantly presented low and few unclear bias risk. The certainty of evidence was high or moderate between placebo comparison, while the precision of drug comparison varied considerably. Moreover, primary endpoints were evaluated at 12, 16, or 24 weeks post-baseline. In order to assess the efficacy and safety of IL-17, IL-12/23, and IL-23 inhibitors for the treatment of PsA, we conducted this NMA to analyze the efficacy ranking and indirect evidence. We found that IL-17 inhibitors (bimekizumab, brodalumab, secukinumab, and ixekizumab), IL-23 inhibitors (guselkumab, tildrakizumab, and risankizumab), and IL-12/23 inhibitors (ustekinumab) were more efficacious in achieving ACR20, ACR50, ACR70, and MDA responses compared to placebo. For ACR20 responses, bimekizumab 160 mg Q4W demonstrated greater efficacy compared with brodalumab (140 mg Q2W and 210 mg Q2W), guselkumab (100 mg Q4W and 100 mg Q8W), ixekizumab (80 mg Q2W and 80 mg Q4W), risankizumab 150 mg, secukinumab (150 mg Q4W and 300 mg Q4W), and ustekinumab (45 mg Q12W and 90 mg Q12W). Additionally, secukinumab 300 mg Q4W showed superior efficacy to ustekinumab 45 mg Q12W. Based on SUCRA values, the treatments ranked from high to low in efficacy were as follows: bimekizumab 160 mg Q4W (SUCRA, 98.6%), secukinumab 300 mg Q4W (SUCRA, 80.3%), and guselkumab 100 mg Q8W (SUCRA, 66.3%). The effect sizes of these comparisons further validated the SUCRA rankings, indicating that the observed differences correspond to clinically meaningful benefits. Subgroup analyses revealed that bimekizumab and guselkumab exhibited favorable efficacy in achieving ACR20 responses among patients with prior anti-TNF exposure. In the anti-TNF-naïve subgroup, bimekizumab and secukinumab 300 mg Q4W showed robust efficacy. A notable deviation from the primary results was observed, with tildrakizumab demonstrating promising efficacy in this subgroup. For ACR50 responses, bimekizumab 160 mg Q4W demonstrated superior efficacy compared with guselkumab (100 mg Q4W and 100 mg Q8W), risankizumab 150 mg, and ustekinumab (45 mg Q12W and 90 mg Q12W). Secukinumab 300 mg Q4W also showed statistically significant superiority over guselkumab 100 mg Q8W and ustekinumab 45 mg Q12W. Although bimekizumab 160 mg Q4W had a higher SUCRA value than secukinumab 300 mg Q4W, no statistically significant difference was observed between these two treatments. Subgroup analyses indicated that bimekizumab 160 mg Q4W, ixekizumab 80 mg Q4W, and tildrakizumab 200 mg Q4W all exhibited favorable efficacy. For ACR70 responses, bimekizumab 160 mg Q4W demonstrated greater efficacy compared with risankizumab 150 mg. However, subgroup analyses revealed that ixekizumab exhibited superior efficacy relative to bimekizumab based on the SUCRA. For MDA, bimekizumab 160 mg Q4W demonstrated superior efficacy to risankizumab 150 mg. Ixekizumab 80 mg Q4W demonstrated superior efficacy to risankizumab 150 mg. Although SUCRA values provided a ranking of treatments based on efficacy for ACR70 and MDA, the minor numerical differences observed are unlikely to be clinically meaningful, as no statistically significant differences were detected between these treatments. No significant differences were observed in ACR20, ACR50, ACR70, or MDA responses between secukinumab 150 mg Q4W and 300 mg Q4W, between ixekizumab 80 mg Q2W and 80 mg Q4W, between ustekinumab 45 mg Q12W and 90 mg Q12W, between guselkumab 100 mg Q4W and 100 mg Q8W, or between brodalumab 140 mg Q2W and 210 mg Q2W. Therefore, for these agents, higher-dose/more frequent regimens showed no advantage over lower-dose/less frequent counterparts, with implications for clinical decision-making. The direct comparison showed that guselkumab 100 Q4W and 100 mg Q8W had no clinically meaningful differences between dosing Q4W and Q8W up to week 24 (35). No distinct clinically meaningful differences were noted between ixekizumab dosing schedules of Q4W versus Q2W regarding arthritis outcomes, thereby validating our study findings (26). Based on efficacy endpoints, bimekizumab, secukinumab, and ixekizumab demonstrated superior short-term efficacy. Subgroup analyses also indicated that these treatments exhibited favorable efficacy rankings in both joint outcomes and disease activity assessments for PsA, regardless of whether patients were anti-TNF-naïve or had prior anti-TNF exposure. Notably, subgroup analyses indicated that tildrakizumab may represent a promising therapeutic option for PsA, and more RCTs are warranted to further investigate its efficacy. Our results align with those of previous meta-analyses that also support the idea that IL-17 and IL-23 inhibitors have demonstrated efficacy in ameliorating joint disease activity among patients with PsA, accompanied by a safety profile that is considered acceptable (16, 46). However, these articles did not analyze which inhibitors demonstrated optimal therapeutic efficacy in PsA. To our knowledge, our study is the first NMA to evaluate the optimal therapeutic efficacy of IL-17, IL-12, or IL-12/23 inhibitors for the treatment of PsA. A study evaluating and comparing the efficacy, safety, and tolerability of IL-6, IL-12/23, and IL-17 inhibitors in patients with PsA was conducted by Wu et al. (47). They reported that secukinumab was identified as the optimal short-term treatment for peripheral PsA in both safety and efficacy profiles, when compared with other novel biologics targeting the IL-6, IL-12/23, and IL-17 signaling pathways. These findings are consistent with our results, which demonstrate that secukinumab exhibits favorable efficacy in the treatment of PsA. A head-to-head monotherapy comparative study found that secukinumab did not reach statistical significance for superiority over adalimumab in the primary endpoint of ACR20 response at week 52 (48). Another direct comparison demonstrated that ixekizumab outperformed adalimumab in achieving concurrent improvement in both joint and skin manifestations (ACR50 and PASI100) among patients with PsA (49). These head-to-head comparative studies collectively confirm the efficacy and safety of IL-17, IL-12, and IL-12/23 inhibitors in the treatment of PsA. Additionally, we underscored the evidentiary certainty by implementing the CINeMA methodology to appraise evidence quality, complemented by tabular presentation of results.

In terms of safety outcomes, we found that there were no differences compared to placebo except for bimekizumab. In terms of SAEs, no significant difference was observed between IL-17, IL-12, or IL-12/23 inhibitors and placebo. The results of another study suggested that IL-17 and IL-23 inhibitors demonstrate favorable tolerability alongside robust safety profiles (50). An additional systematic review also suggested that patients with psoriasis treated with IL-17 or IL-23 inhibitors have generally low short-term infection risks and long-term malignancy incidences (51). Furthermore, statistically significant differences in the incidence of AEs and nasopharyngitis were observed between the bimekizumab and placebo groups. Notably, the risk of nasopharyngitis associated with bimekizumab warrants special attention. This finding is consistent with the work of Mahmoud et al., who also reported a significantly increased risk of nasopharyngitis with bimekizumab compared to placebo (52). Collectively, these observations suggest that the safety profile of bimekizumab may raise considerations regarding its clinical use.

Findings from the 3-year PsABio real-world study indicated comparable treatment persistence between ustekinumab and TNFi, with ustekinumab associated with a lower incidence of AEs (53). Two long-term RCTs showed that risankizumab was well-tolerated and effective over 52-week treatment periods (35, 36). A real-world cohort study reported sustained high long-term drug survival and efficacy of secukinumab, alongside a favorable safety profile, after 42 months (54). Additionally, a study involving 1,401 patients with PsA with over 2,000 patient-years of ixekizumab exposure confirmed its long-term safety, with an adjusted AE incidence of 50.3 per 100 patient-years and most events being mild to moderate in severity (55). Bimekizumab also exhibited favorable efficacy and safety, with rapid onset of action and sustained efficacy through week 48 (23). Consistent with our study, which demonstrated good efficacy and safety within 24 weeks, these long-term data collectively confirm the superior sustained efficacy and safety profiles of IL-17, IL-12, and IL-12/23 inhibitors. Antonazzo et al. reported that PsA remains associated with a substantial global economic burden, with therapies—particularly biological agents—serving as the primary cost driver. Among these therapies, bimekizumab and ixekizumab provide the most significant economic benefits (56). Similarly, Sigurdardottir et al. noted that bimekizumab was cost-effective compared with most available treatments, including other IL-17A, IL-23, and JAK inhibitors, for PsA management in Sweden, irrespective of prior TNF exposure (57). Sbidian et al. showed that to achieve PASI 90 and PASI 75 in psoriasis treatment, the most effective agents vs. placebo—per SUCRA rankings with high-certainty evidence for all—were infliximab, bimekizumab, ixekizumab, and risankizumab (58). Therefore, from a cost-effectiveness perspective, bimekizumab and ixekizumab emerge as superior therapeutic options.

Despite the robust evidentiary basis and methodological rigor, this NMA is not without limitations. First, heterogeneity (as measured by *I* ^2^ and *τ*²) was observed for most outcomes. According to our CINeMA ratings, confidence in findings from most pairwise drug comparisons was low, often attributable to factors such as imprecision, heterogeneity, or incoherence. Additionally, funnel plots suggested potential publication bias. Potential publication bias for ACR20 and ACR70 may stem from overrepresentation of studies with positive findings (e.g., significant efficacy) and underrepresentation of those with negative or small effect results, which could overestimate pooled effects—exaggerating observed drug efficacy advantages and amplifying inter-drug differences that require cautious interpretation. In contrast, ACR50 and AEs showed no significant bias, likely because the higher significance of ACR50 threshold facilitates the publication of negative results, while regulatory requirements mandate AE data disclosure regardless of outcomes. Collectively, these issues indicate that the risk of bias in many studies may be largely associated with prior biologic exposure and the pooling of outcomes assessed at heterogeneous time points. To verify result reliability, we conducted a sensitivity analysis for ACR20 by sequentially excluding each time point. Efficacy outcomes from this analysis were consistent with the primary results, thereby reinforcing the validity of our conclusions. Furthermore, subgroup analyses also revealed that bimekizumab, secukinumab, and ixekizumab maintained favorable efficacy irrespective of patients’ anti-TNF status (anti-TNF-naïve or with prior exposure). All these analyses contribute to enhancing the reliability of our conclusions. Second, the analytical dataset was exclusively derived from RCTs, a design that may not fully mirror real-world patient demographics. Third, despite being placebo-controlled during induction, maintenance phases lacked placebo controls, complicating data extraction and analysis. Thus, we assessed primary endpoints at induction completion (12, 16, or 24 weeks). Whether biologics ultimately improve the quality of life of patients with PsA remains unclear and requires further study. Fourth, the study findings are solely based on the current published literature, meaning conclusions may evolve as new evidence emerges. Ongoing follow-up and updates within a 3-year period are planned to incorporate emerging research.

## 5 Conclusion

IL-17 inhibitors (bimekizumab, brodalumab, secukinumab, and ixekizumab), IL-23 inhibitors (guselkumab, tildrakizumab, and risankizumab), and IL-12/23 inhibitors (ustekinumab) have emerged as promising therapeutic strategies for PsA. In this NMA, IL-17 inhibitors have shown superior efficacy, especially bimekizumab, secukinumab, and ixekizumab. However, the clinical safety profile of bimekizumab is less favorable relative to other biological agents. Simultaneously, secukinumab and ixekizumab emerge as high-efficacy, low-risk therapeutic options. These findings suggest a novel clinical paradigm for PsA intervention. Moreover, tildrakizumab may represent a promising therapeutic option for PsA. However, prospective head-to-head clinical trials are required to validate the long-term efficacy and safety of these agents.

## Data Availability

The original contributions presented in the study are included in the article/**Supplementary Material**. Further inquiries can be directed to the corresponding author.
