# Supplementary material for: Efficacy and safety of IL-17, IL-12/23, and IL-23 inhibitors for psoriatic arthritis: a network meta-analysis of randomized controlled trials
Source: Front Immunol. 2025 Sep 18;16:1654343. doi: 10.3389/fimmu.2025.1654343 (PMC12489988; doi:10.3389/fimmu.2025.1654343)
Supplement: Supplementary file 1 [file Table1.docx]

Supplementary Material

**Supplementary Table S1.** PRISMA NMA Checklist of Items to Include When Reporting A Systematic Review Involving a Network Meta-analysis

| **Section/Topic** | **Item #** | **Checklist Item** | **Reported on Page #** |
| --- | --- | --- | --- |
| **TITLE** |  |  |  |
| Title | 1 | Identify the report as a systematic review *incorporating a network meta-analysis (or related form of meta-analysis).* | **1** |
|  |  |  |  |
| **ABSTRACT** |  |  |  |
| Structured summary | 2 | Provide a structured summary including, as applicable:  **Background:** main objectives  **Methods:** data sources; study eligibility criteria, participants, and interventions; study appraisal; and *synthesis methods, such as network meta-analysis.*  **Results:** number of studies and participants identified; summary estimates with corresponding confidence/credible intervals; *treatment rankings may also be discussed. Authors may choose to summarize pairwise comparisons against a chosen treatment included in their analyses for brevity.*  **Discussion/Conclusions:** limitations; conclusions and implications of findings.  **Other:** primary source of funding; systematic review registration number with registry name. | 1-2 |
|  |  |  |  |
| **INTRODUCTION** |  |  |  |
| Rationale | 3 | Describe the rationale for the review in the context of what is already known*, including mention of why a network meta-analysis has been conducted.* | **2** |
| Objectives | 4 | Provide an explicit statement of questions being addressed, with reference to participants, interventions, comparisons, outcomes, and study design (PICOS). | 2 |
|  |  |  |  |
| **METHODS** |  |  |  |
| Protocol and registration | 5 | Indicate whether a review protocol exists and if and where it can be accessed (e.g., Web address); and, if available, provide registration information, including registration number. | 3 |
| Eligibility criteria | 6 | Specify study characteristics (e.g., PICOS, length of follow-up) and report characteristics (e.g., years considered, language, publication status) used as criteria for eligibility, giving rationale. *Clearly describe eligible treatments included in the treatment network, and note whether any have been clustered or merged into the same node (with justification).* | **3** |
| Information sources | 7 | Describe all information sources (e.g., databases with dates of coverage, contact with study authors to identify additional studies) in the search and date last searched. | 3 |
| Search | 8 | Present full electronic search strategy for at least one database, including any limits used, such that it could be repeated. | 3 Supplementary Table S2 |
| Study selection | 9 | State the process for selecting studies (i.e., screening, eligibility, included in systematic review, and, if applicable, included in the meta-analysis). | 3 Table 1 |
| Data collection process | 10 | Describe method of data extraction from reports (e.g., piloted forms, independently, in duplicate) and any processes for obtaining and confirming data from investigators. | 3 |
| Data items | 11 | List and define all variables for which data were sought (e.g., PICOS, funding sources) and any assumptions and simplifications made. | 3-4 |
| **Geometry of the network** | **S1** | Describe methods used to explore the geometry of the treatment network under study and potential biases related to it. This should include how the evidence base has been graphically summarized for presentation, and what characteristics were compiled and used to describe the evidence base to readers. | **4** |
| Risk of bias within individual studies | 12 | Describe methods used for assessing risk of bias of individual studies (including specification of whether this was done at the study or outcome level), and how this information is to be used in any data synthesis. | 3-4 |
| Summary measures | 13 | State the principal summary measures (e.g., risk ratio, difference in means). *Also describe the use of additional summary measures assessed, such as treatment rankings and surface under the cumulative ranking curve (SUCRA) values, as well as modified approaches used to present summary findings from meta-analyses.* | 4 |
| Planned methods of analysis | 14 | Describe the methods of handling data and combining results of studies for each network meta-analysis. This should include, but not be limited to:   - *Handling of multi-arm trials;* - *Selection of variance structure;* - *Selection of prior distributions in Bayesian analyses; and* - *Assessment of model fit.* | 4 |
| **Assessment of Inconsistency** | **S2** | Describe the statistical methods used to evaluate the agreement of direct and indirect evidence in the treatment network(s) studied. Describe efforts taken to address its presence when found. | 4 |
| Risk of bias across studies | 15 | Specify any assessment of risk of bias that may affect the cumulative evidence (e.g., publication bias, selective reporting within studies). | **4** |
| Additional analyses | 16 | Describe methods of additional analyses if done, indicating which were pre-specified. This may include, but not be limited to, the following:   - Sensitivity or subgroup analyses; - Meta-regression analyses; - *Alternative formulations of the treatment network; and* - *Use of alternative prior distributions for Bayesian analyses (if applicable).* | **4** |
|  |  |  |  |
| **RESULTS†** |  |  |  |
| Study selection | 17 | Give numbers of studies screened, assessed for eligibility, and included in the review, with reasons for exclusions at each stage, ideally with a flow diagram. | 5 Figure 1 |
| **Presentation of network structure** | **S3** | Provide a network graph of the included studies to enable visualization of the geometry of the treatment network. | 5 Figure 3 |
| **Summary of network geometry** | **S4** | Provide a brief overview of characteristics of the treatment network. This may include commentary on the abundance of trials and randomized patients for the different interventions and pairwise comparisons in the network, gaps of evidence in the treatment network, and potential biases reflected by the network structure. | **5** |
| Study characteristics | 18 | For each study, present characteristics for which data were extracted (e.g., study size, PICOS, follow-up period) and provide the citations. | 5 Table 1 |
| Risk of bias within studies | 19 | Present data on risk of bias of each study and, if available, any outcome level assessment. | 5 Figure 2 and Supplementary Table S3 |
| Results of individual studies | 20 | For all outcomes considered (benefits or harms), present, for each study: 1) simple summary data for each intervention group, and 2) effect estimates and confidence intervals. *Modified approaches may be needed to deal with information from larger networks.* | 5-7 |
| Synthesis of results | 21 | Present results of each meta-analysis done, including confidence/credible intervals. *In larger networks, authors may focus on comparisons versus a particular comparator (e.g. placebo or standard care), with full findings presented in an appendix. League tables and forest plots may be considered to summarize pairwise comparisons.* If additional summary measures were explored (such as treatment rankings), these should also be presented. | 5-7 |
| **Exploration for inconsistency** | **S5** | Describe results from investigations of inconsistency. This may include such information as measures of model fit to compare consistency and inconsistency models, *P* values from statistical tests, or summary of inconsistency estimates from different parts of the treatment network. | **5-7** |
| Risk of bias across studies | 22 | Present results of any assessment of risk of bias across studies for the evidence base being studied. | 8 |
| Results of additional analyses | 23 | Give results of additional analyses, if done (e.g., sensitivity or subgroup analyses, meta-regression analyses*, alternative network geometries studied, alternative choice of prior distributions for Bayesian analyses,* and so forth). | **7-8** |
|  |  |  |  |
| **DISCUSSION** |  |  |  |
| Summary of evidence | 24 | Summarize the main findings, including the strength of evidence for each main outcome; consider their relevance to key groups (e.g., healthcare providers, users, and policy-makers). | 8 |
| Limitations | 25 | Discuss limitations at study and outcome level (e.g., risk of bias), and at review level (e.g., incomplete retrieval of identified research, reporting bias). *Comment on the validity of the assumptions, such as transitivity and consistency. Comment on any concerns regarding network geometry (e.g., avoidance of certain comparisons).* | 10-11 |
| Conclusions | 26 | Provide a general interpretation of the results in the context of other evidence, and implications for future research. | 11 |
|  |  |  |  |
| **FUNDING** |  |  |  |
| Funding | 27 | Describe sources of funding for the systematic review and other support (e.g., supply of data); role of funders for the systematic review. This should also include information regarding whether funding has been received from manufacturers of treatments in the network and/or whether some of the authors are content experts with professional conflicts of interest that could affect use of treatments in the network. | **12** |

PICOS = population, intervention, comparators, outcomes, study design.

* Text in italics indicateS wording specific to reporting of network meta-analyses that has been added to guidance from the PRISMA statement.

† Authors may wish to plan for use of appendices to present all relevant information in full detail for items in this section.

| **Supplementary Table S2.Literature Search Strategy** | |
| --- | --- |
| **Pubmed** | (("Psoriasis"[Mesh]) OR ("Arthritis, Psoriatic"[Mesh])) AND (("Interleukin-17"[Mesh]) OR ("Interleukin-23"[Mesh])OR ("Interleukin-12"[Mesh]))  Final results: 2344 |
| **Web of Science** | (TS=(psoriasis) OR TS=(psoriatic arthritis)) AND (TS=(IL-17 inhibitor*) OR TS=(IL-23 inhibitor*)OR TS=(IL-12 inhibitor*))  Final results: 1541 |
| **ClinicalTrials.gov** | Psoriasis OR Psoriatic arthritis (condition/disease) + Brodalumab (intervention/treatment) + filter (with results): 14  Psoriasis OR Psoriatic arthritis (condition/disease) + Ixekizumab (intervention/treatment) + filter (with results): 22  Psoriasis OR Psoriatic arthritis (condition/disease) + Secukinumab (intervention/treatment) + filter (with results): 64  Psoriasis OR Psoriatic arthritis (condition/disease) + Bimekizumab (intervention/treatment) + filter (with results): 16  Psoriasis OR Psoriatic arthritis (condition/disease) + Guselkumab (intervention/treatment) + filter (with results): 15  Psoriasis OR Psoriatic arthritis (condition/disease) + Risankizumab (intervention/treatment) + filter (with results): 19  Psoriasis OR Psoriatic arthritis (condition/disease) + Tildrakizumab (intervention/treatment) + filter (with results): 8  Psoriasis OR Psoriatic arthritis (condition/disease) + Ustekinumab (intervention/treatment) + filter (with results): 38  Final results: 196 |
| **Embase** | ((exp interleukin 1 receptor blocking agent/ or exp interleukin 17/ or exp interleukin 23/or exp interleukin 12)OR( exp ustekinumab/ or Ustekinumab.ti,ab. or exp secukinumab/ or secukinumab.ti,ab. or exp bimekizumab/ or bimekizumab.ti,ab. or exp guselkumab/ or guselkumab.ti,ab. or exp risankizumab/ or risankizumab.ti,ab. or exp ixekizumab/ or ixekizumab.ti,ab. or exp brodalumab/ or brodalumab.ti,ab. or exp tildrakizumab/ or tildrakizumab.ti,ab.)))AND (exp psoriatic arthritis/ or psoriatic arthritis.ti,ab.)  Final results: 1636 |

# Supplementary Table S3. Risk of bias

| **Study** | **Random sequence generation** | **Allocation concealment** | **Blinding of participants and personnel** | **Blinding of outcome assessment** | **Incomplete outcome data** | **Other source of bias** |
| --- | --- | --- | --- | --- | --- | --- |
| Ritchlin et al, 2020 | Low risk | Low risk | Low risk | Unclear risk | Low risk | Low risk |
| Merola et al, 2023 | Low risk | Low risk | Low risk | Unclear risk | Low risk | Low risk |
| McInnes et al, 2023 | Low risk | Low risk | Low risk | Unclear risk | Low risk | Low risk |
| Mease et al, 2021a | Low risk | Low risk | Low risk | Unclear risk | Low risk | Low risk |
| Mease et al, 2021b | Low risk | Low risk | Low risk | Unclear risk | Unclear risk | Low risk |
| Mease et al, 2017 | Low risk | Low risk | Low risk | Low risk | Low risk | Low risk |
| Nash et al, 2017 | Low risk | Low risk | Low risk | Low risk | Low risk | Low risk |
| Nash et al, 2018 | Low risk | Low risk | Low risk | Unclear risk | Low risk | Low risk |
| Kivitz et al, 2019 | Low risk | Low risk | Low risk | Unclear risk | Low risk | Low risk |
| McInnes et al, 2015 | Low risk | Low risk | Low risk | Unclear risk | Low risk | Low risk |
| Mease et al, 2018 | Low risk | Low risk | Low risk | Low risk | Low risk | Low risk |
| Baraliakos et al, 2021 | Low risk | Low risk | Low risk | Unclear risk | Low risk | Low risk |
| Mease PJ et al 2021 | Low risk | Low risk | Low risk | Unclear risk | Low risk | Low risk |
| Kristensen et al, 2022 | Low risk | Low risk | Low risk | Unclear risk | Low risk | Low risk |
| Ostor et al, 2022 | Low risk | Low risk | Low risk | Unclear risk | Low risk | Low risk |
| Mease et al, 2020 | Low risk | Low risk | Low risk | Low risk | Low risk | Low risk |
| Deodhar et al, 2020 | Low risk | Low risk | Low risk | Low risk | Low risk | Low risk |
| Coates et al, 2022 | Low risk | Low risk | Low risk | Low risk | Low risk | Low risk |
| Deodhar et al, 2018 | Low risk | Low risk | Low risk | Low risk | Low risk | Low risk |
| Ritchlin et al, 2014 | Low risk | Low risk | Low risk | Unclear risk | Low risk | Low risk |
| McInnes et al, 2013 | Low risk | Low risk | Low risk | Low risk | Low risk | Low risk |
| Gottlieb et al, 2009 | Low risk | Low risk | Low risk | Low risk | Low risk | Low risk |

**Supplementary Table S4 Node splitting test for inconsistency**

| **Outcome** | **Side** | **Direct** | | **Indirect** | | **Difference** | |  |
| --- | --- | --- | --- | --- | --- | --- | --- | --- |
|  |  | Coef. | Std. Err. | Coef. | Std. Err. | Coef. | Std. Err. | ***P* > \|z** |
| ACR20 | A VS. B | - | - | - | - | - | - | - |
|  | A VS. C | - | - | - | - | - | - | - |
|  | A VS. D | - | - | - | - | - | - | - |
|  | A VS. E | - | - | - | - | - | - | - |
|  | A VS. F | - | - | - | - | - | - | - |
|  | A VS. G | 1.538 | 0.124 | 1.151 | 0.686 | 0.387 | 0.701 | 0.581 |
|  | A VS. H | - | - | - | - | - | - | - |
|  | A VS. I | - | - | - | - | - | - | - |
|  | A VS. J | - | - | - | - | - | - | - |
|  | A VS. K | 1.40 | .176 | 1.48 | 0.589 | -0.818 | 0.618 | 0.895 |
|  | A VS. L | - | - | - | - | - | - | - |
|  | A VS. M | 0.987 | 0.193 | 1.59 | 0.905 | -0.603 | 0.931 | 0.0517 |
|  | A VS. N | - | - | - | - | - | - | - |
|  | C VS. D | - | - | - | - | - | - | - |
|  | E VS. F | - | - | - | - | - | - | - |
|  | G VS. H | -0.211 | 0.127 | -0.598 | 0.689 | 0.387 | 0.701 | 0.581 |
|  | K VS. L | -0.102 | 0.169 | -0.020 | 0.593 | -0.818 | 0.617 | 0.895 |
|  | M VS. N | 0.185 | 0.177 | 0.788 | 0.914 | -0.603 | 0.931 | 0.517 |
| ACR50 | A VS. B | - | - | - | - | - | - | - |
|  | A VS. C | - | - | - | - | - | - | - |
|  | A VS. D | - | - | - | - | - | - | - |
|  | A VS. E | - | - | - | - | - | - | - |
|  | A VS. F | - | - | - | - | - | - | - |
|  | A VS. G | 1.901 | 0.216 | 1.708 | 1.029 | 0.1991 | 1.067 | 0.852 |
|  | A VS. H | - | - | - | - | - | - | - |
|  | A VS. I | - | - | - | - | - | - | - |
|  | A VS. J | - | - | - | - | - | - | - |
|  | A VS. K | 1.316 | 0.236 | 1.993 | 0.841 | -0.676 | 0.891 | 0.448 |
|  | A VS. L | - | - | - | - | - | - | - |
|  | A VS. M | 1.186 | 0.286 | 1.306 | 1.209 | -0.119 | 1.26 | 0.925 |
|  | A VS. N | - | - | - | - | - | - | - |
|  | C VS. D | - | - | - | - | - | - | - |
|  | E VS. F | - | - | - | - | - | - | - |
|  | G VS. H | -0.299 | 0.180 | -0.4989 | -1.050 | 0.199 | 1.067 | 1.067 |
|  | K VS. L | -0.154 | 0.202 | 0.522 | 0.522 | -0.677 | 0.891 | 0.891 |
|  | M VS. N | -0.220 | 0.234 | 0.339 | 0.339 | -0.119 | 1.262 | 1.262 |
| ACR70 | A VS. B | - | - | - | - | - | - | - |
|  | A VS. C | - | - | - | - | - | - | - |
|  | A VS. D | - | - | - | - | - | - | - |
|  | A VS. E | - | - | - | - | - | - | - |
|  | A VS. F | - | - | - | - | - | - | - |
|  | A VS. G | 1.929 | 0.441 | 2.71 | 2.334 | -0.780 | 2.417 | 0.747 |
|  | A VS. H | - | - | - | - | - | - | - |
|  | A VS. I | - | - | - | - | - | - | - |
|  | A VS. J | - | - | - | - | - | - | - |
|  | A VS. K | 1.37 | 0.389 | 2.87 | 1.686 | -1.50 | 1.754 | 0.393 |
|  | A VS. L | - | - | - | - | - | - | - |
|  | A VS. M | 1.153 | 0.402 | 4.20 | 3.084 | -3.047 | 3.121 | 0.329 |
|  | A VS. N | - | - | - | - | - | - | - |
|  | C VS. D | - | - | - | - | - | - | - |
|  | E VS. F | - | - | - | - | - | - | - |
|  | G VS. H | -0.049 | 0.331 | 0.730 | 2.392 | -0.780 | 2.412 | 0.747 |
|  | K VS. L | -0.038 | 0.337 | 1.461 | 1.720 | -1.498 | 1.754 | 0.393 |
|  | M VS. N | 0.184 | 0.353 | 3.232 | 3.102 | -3.048 | 3.121 | 0.329 |
| AE | A VS. B | - | - | - | - | - | - | - |
|  | A VS. C | - | - | - | - | - | - | - |
|  | A VS. D | - | - | - | - | - | - | - |
|  | A VS. E | - | - | - | - | - | - | - |
|  | A VS. F | - | - | - | - | - | - | - |
|  | A VS. G | - | - | - | - | - | - | - |
|  | A VS. H | - | - | - | - | - | - | - |
|  | A VS. I | - | - | - | - | - | - | - |
|  | A VS. J | - | - | - | - | - | - | - |
|  | A VS. K | 0.091 | 0.148 | -0.245 | 0.487 | 0.335 | 0.510 | 0.511 |
|  | A VS. L | - | - | - | - | - | - | - |
|  | A VS. M | 0.225 | 0.165 | -0.236 | 0.739 | 0.462 | 0.757 | 0.541 |
|  | A VS. N | - | - | - | - | - | - | - |
|  | C VS. D | - | - | - | - | - | - | - |
|  | E VS. F | - | - | - | - | - | - | - |
|  | G VS. H | - | - | - | - | - | - | - |
|  | K VS. L | -0.301 | 0.146 | -0.365 | 0.489 | 0.335 | 0.510 | 0.511 |
|  | M VS. N | 0.225 | 0.165 | -0.556 | 0.739 | 0.462 | 0.756 | 0.541 |
| SAEs | A VS. B | - | - | - | - | - | - | - |
|  | A VS. C | - | - | - | - | - | - | - |
|  | A VS. D | - | - | - | - | - | - | - |
|  | A VS. E | - | - | - | - | - | - | - |
|  | A VS. F | - | - | - | - | - | - | - |
|  | A VS. G | - | - | - | - | - | - | - |
|  | A VS. H | - | - | - | - | - | - | - |
|  | A VS. I | - | - | - | - | - | - | - |
|  | A VS. J | - | - | - | - | - | - | - |
|  | A VS. K | -0.194 | 0.481 | 0.943 | 1.558 | -0.962 | 1.576 | 0.541 |
|  | A VS. L | - | - | - | - | - | - | - |
|  | A VS. M | -0.180 | 0.549 | 4.985 | 3.21 | -5.165 | 3.224 | 0.109 |
|  | A VS. N | - | - | - | - | - | - | - |
|  | C VS. D | - | - | - | - | - | - | - |
|  | E VS. F | - | - | - | - | - | - | - |
|  | G VS. H | - | - | - | - | - | - | - |
|  | K VS. L | -0.461 | 0.560 | 0.501 | 1.478 | -0.962 | 1.576 | 0.541 |
|  | M VS. N | -0.499 | 0.613 | 4.667 | 3.170 | -5.165 | 3.224 | 0.109 |

ACR:American College of Rheumatology response; AEs: adverse events; SAEs: serious adverse events; A, PLACEBO; B, bimekizumab 160 mg; C, brodalumab 140mg Q2W; D, brodalumab 210mg Q2W; E, ixekizumab Q4W; F, ixekizumab Q2W; G, secukinumab 300 mg; H, secukinumab 150 mg; I, tildrakizumab 200mg; J, risankizumab 150mg; K, guselkumab 100 mg Q4W; L, guselkumab 100 mg Q8W; M, ustekinumab 45mg Q12W; N, ustekinumab 90mg Q12W.

**Supplementary Figure S1 Global inconsistency**

A ACR20 A ACR50


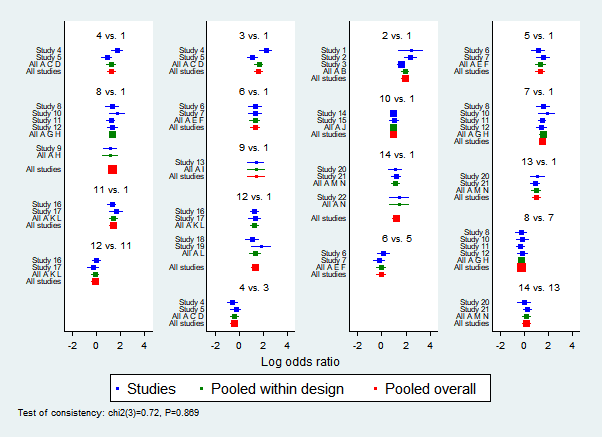

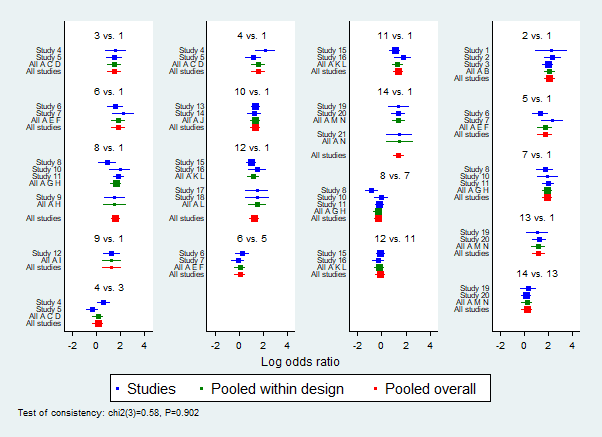


C ACR70 D MDA


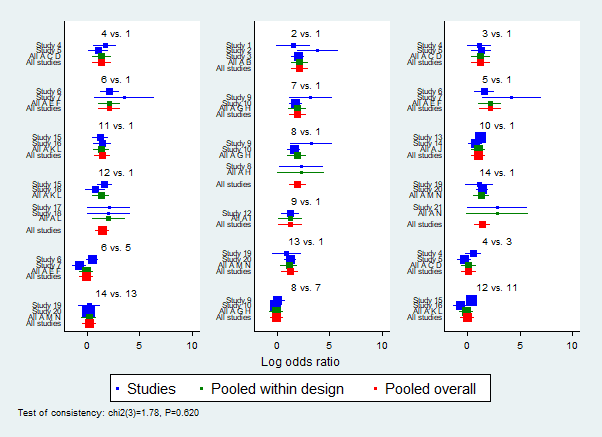

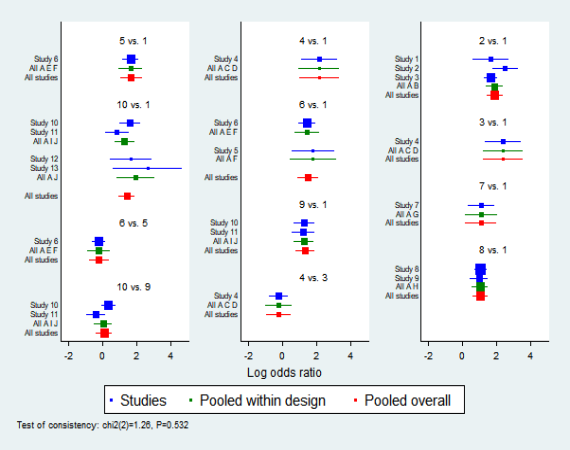


E AEs F SAE


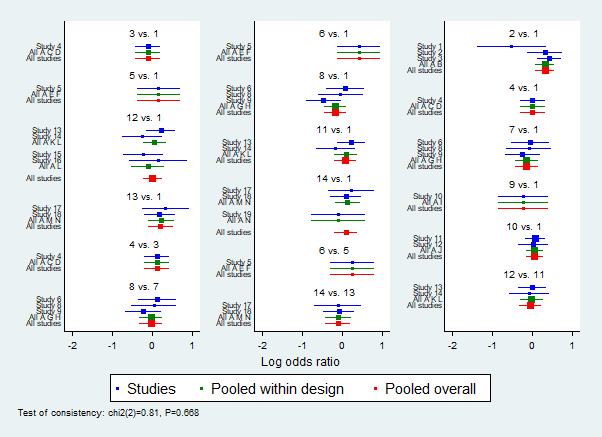

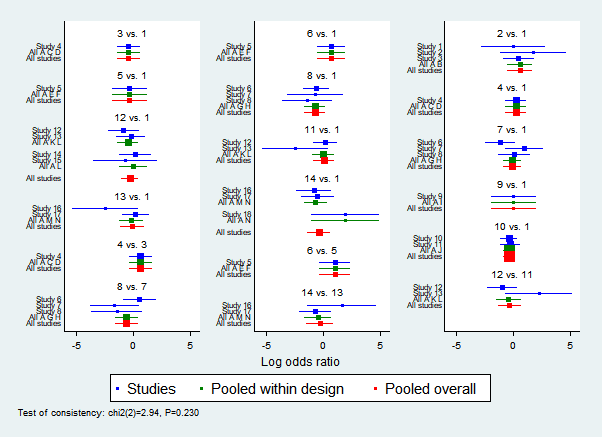


ACR:American College of Rheumatology response;MDA:minimal disease activity; AEs: adverse events; SAEs: serious adverse events.

**Supplementary Figure S2 The results for the NMA compared to placebo**

A ACR20 B ACR50


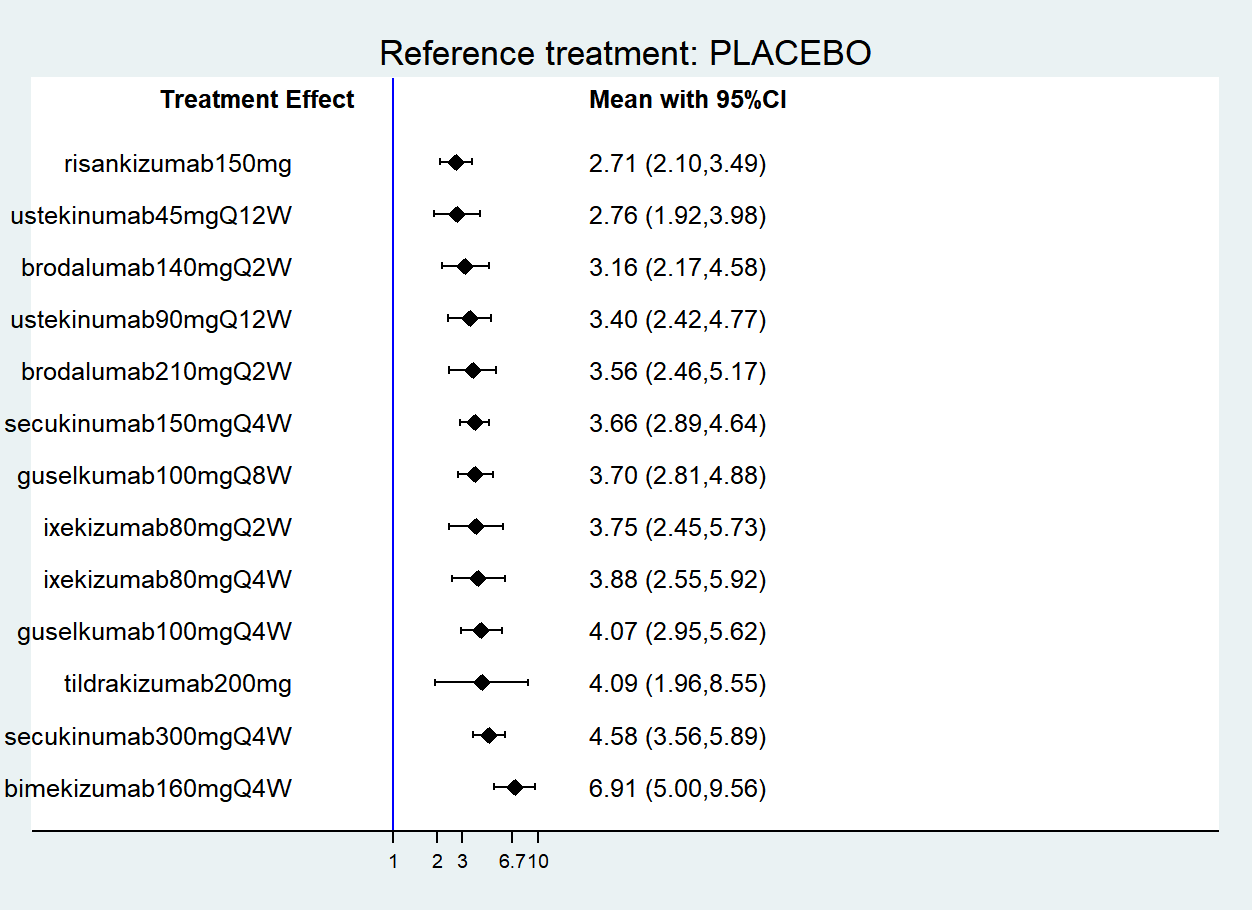

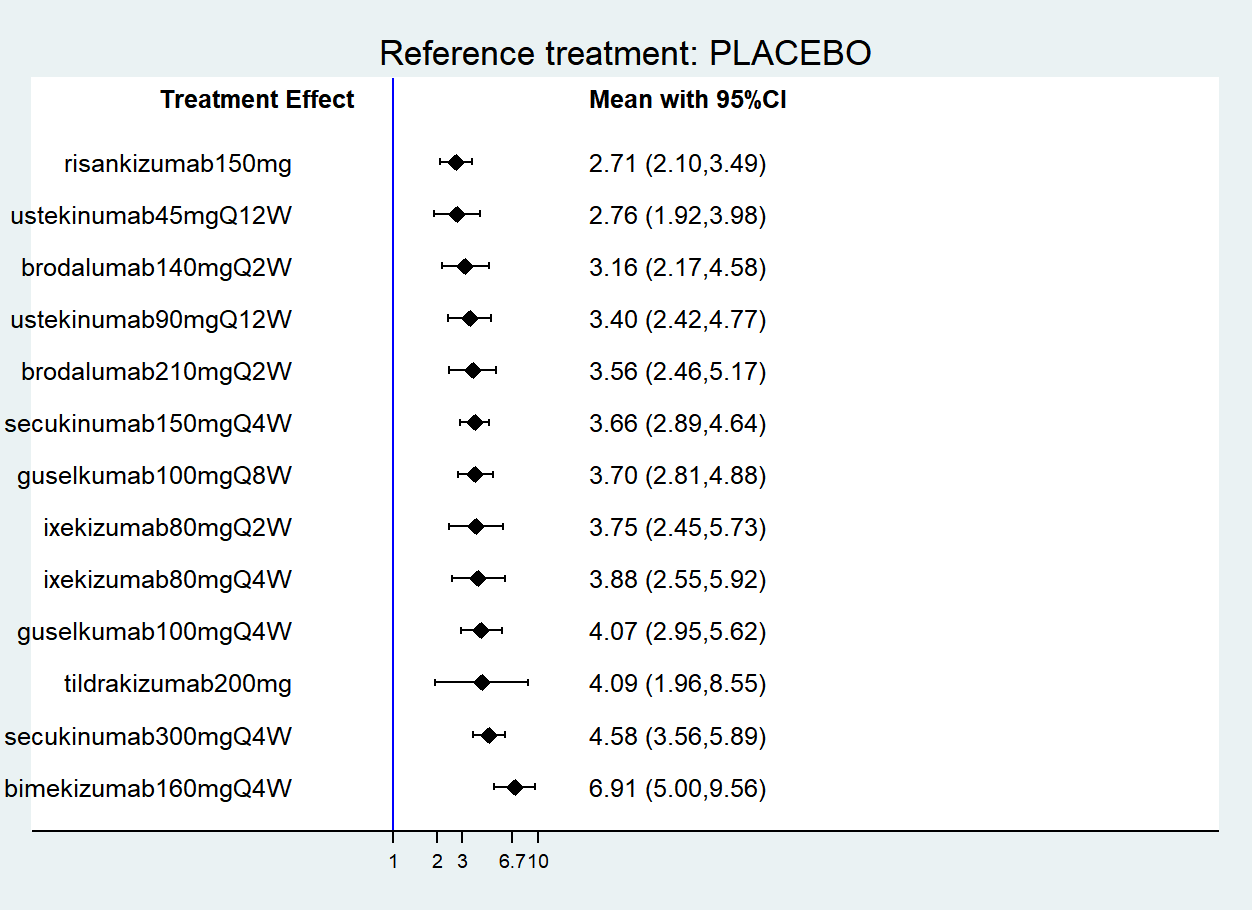


C ACR70 D MDA


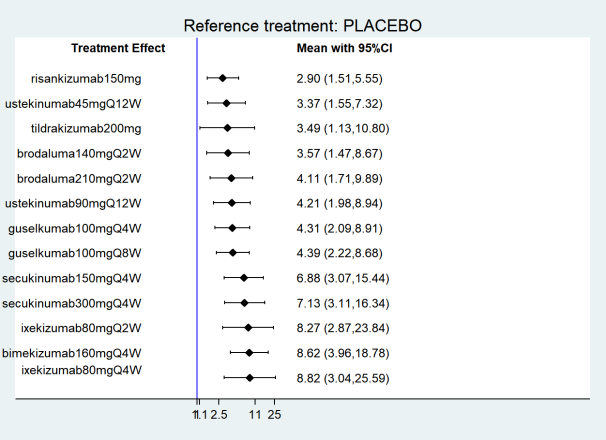

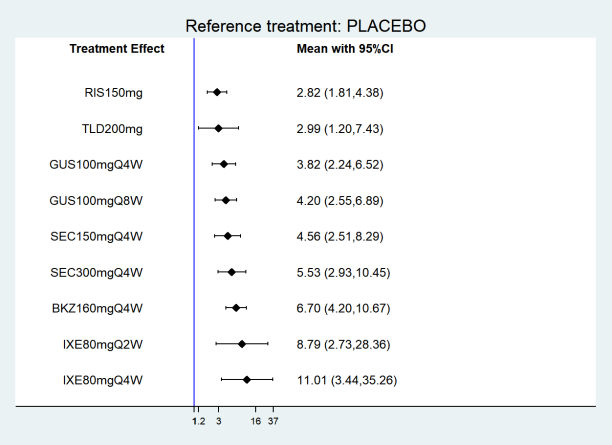


E AEs F SAEs


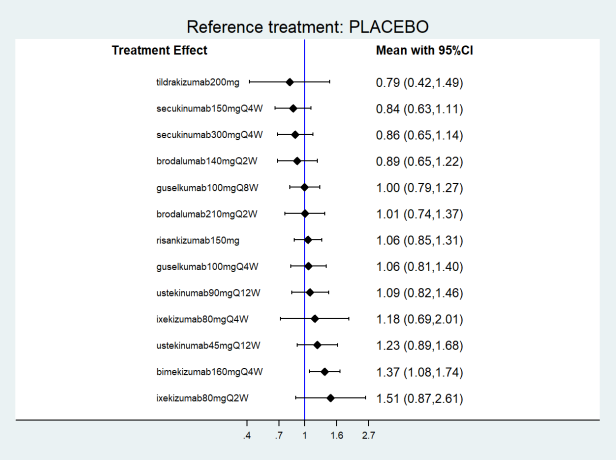

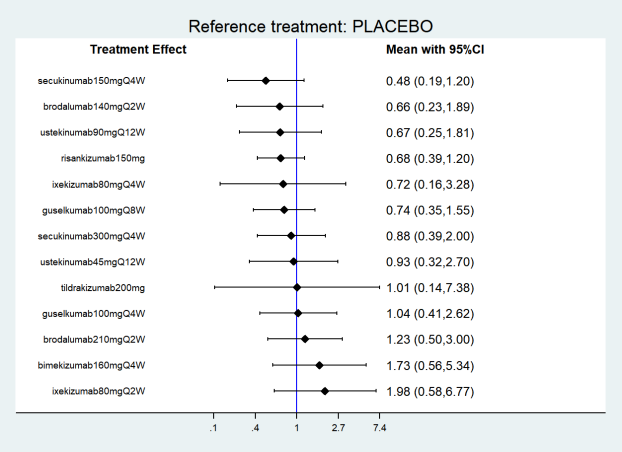


G Nasopharyngitis H Upper respiratory tract infection


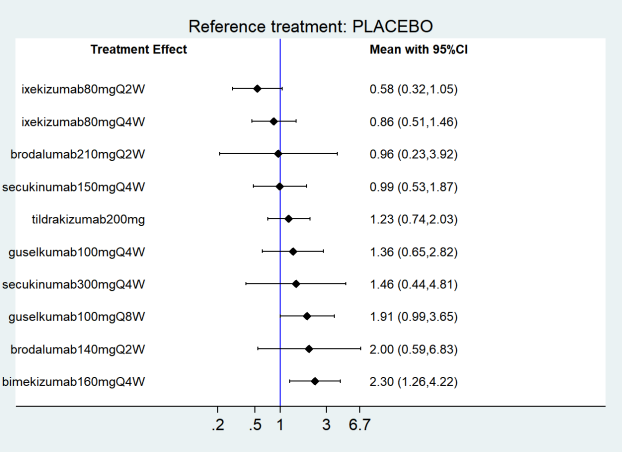

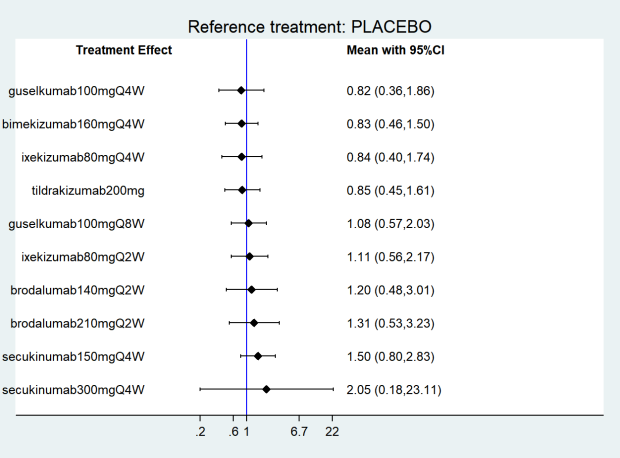


ACR: American College of Rheumatology response; MDA: minimal disease activity; AEs: adverse events; SAEs: serious adverse events; PBO: PLACEBO; BKZ: bimekizumab; BRO: brodalumab; IXE: ixekizumab; SEC: secukinumab; TLD: tildrakizumab; RIS: risankizumab; GUS: guselkumab; UST: ustekinumab.

**Supplementary Table S5: CINeMA ratings for all comparisons of the four outcomes**

| **Comparison** | **Nature of evidence** | **Confidence level** | **Downgrading** |
| --- | --- | --- | --- |
| **ACR20** | | | |
| BKZ160mgQ4W:PLACEBO | mixed | High |  |
| BRO140mgQ2W:BRO210mgQ2W | mixed | Low | Imprecision、Heterogeneity |
| BRO140mgQ2W:PLACEBO | mixed | Low | Serious Incoherence |
| BRO210mgQ2W:PLACEBO | mixed | High |  |
| GUS100mgQ4W:GUS100mgQ8W | mixed | Low | Imprecision、Heterogeneity |
| GUS100mgQ4W:PLACEBO | mixed | High |  |
| GUS100mgQ8W:PLACEBO | mixed | High |  |
| IXE80mgQ2W:IXE80mgQ4W | mixed | Low | Serious Imprecision |
| IXE80mgQ2W:PLACEBO | mixed | High |  |
| IXE80mgQ4W:PLACEBO | mixed | High |  |
| PLACEBO:RIS150mg | mixed | High |  |
| PLACEBO:SEC150mgQ4W | mixed | High |  |
| PLACEBO:SEC300mgQ4W | mixed | High |  |
| PLACEBO:TLD200mg | mixed | High |  |
| PLACEBO:UST45mgQ12W | mixed | High |  |
| PLACEBO:UST90mgQ12W | mixed | High |  |
| SEC150mgQ4W:SEC300mgQ4W | mixed | Moderate | Imprecision |
| UST45mgQ12W:UST90mgQ12W | mixed | Moderate | Imprecision |
| BKZ160mgQ4W:BRO140mgQ2W | indrect | High |  |
| BKZ160mgQ4W:BRO210mgQ2W | indrect | High |  |
| BKZ160mgQ4W:GUS100mgQ4W | indrect | High |  |
| BKZ160mgQ4W:GUS100mgQ8W | indrect | High |  |
| BKZ160mgQ4W:IXE80mgQ2W | indrect | High |  |
| BKZ160mgQ4W:IXE80mgQ4W | indrect | High |  |
| BKZ160mgQ4W:RIS150mg | indrect | High |  |
| BKZ160mgQ4W:SEC150mgQ4W | indrect | High |  |
| BKZ160mgQ4W:SEC300mgQ4W | indrect | Moderate | Heterogeneity |
| BKZ160mgQ4W:TLD200mg | indrect | Low | Serious Imprecision |
| BKZ160mgQ4W:UST45mgQ12W | indrect | High |  |
| BKZ160mgQ4W:UST90mgQ12W | indrect | High |  |
| BRO140mgQ2W:GUS100mgQ4W | indrect | Low | Serious Imprecision |
| BRO140mgQ2W:GUS100mgQ8W | indrect | Low | Serious Imprecision |
| BRO140mgQ2W:IXE80mgQ2W | indrect | Low | Serious Imprecision |
| BRO140mgQ2W:IXE80mgQ4W | indrect | Low | Serious Imprecision |
| BRO140mgQ2W:RIS150mg | indrect | Low | Serious Imprecision |
| BRO140mgQ2W:SEC150mgQ4W | indrect | Low | Serious Imprecision |
| BRO140mgQ2W:SEC300mgQ4W | indrect | Moderate | Imprecision |
| BRO140mgQ2W:TLD200mg | indrect | Low | Serious Imprecision |
| BRO140mgQ2W:UST45mgQ12W | indrect | Low | Serious Imprecision |
| BRO140mgQ2W:UST90mgQ12W | indrect | Low | Serious Imprecision |
| BRO210mgQ2W:GUS100mgQ4W | indrect | Low | Serious Imprecision |
| BRO210mgQ2W:GUS100mgQ8W | indrect | Low | Serious Imprecision |
| BRO210mgQ2W:IXE80mgQ2W | indrect | Low | Serious Imprecision |
| BRO210mgQ2W:IXE80mgQ4W | indrect | Low | Serious Imprecision |
| BRO210mgQ2W:RIS150mg | indrect | Moderate | Imprecision |
| BRO210mgQ2W:SEC150mgQ4W | indrect | Low | Serious Imprecision |
| BRO210mgQ2W:SEC300mgQ4W | indrect | Low | Imprecision、Heterogeneity |
| BRO210mgQ2W:TLD200mg | indrect | Low | Serious Imprecision |
| BRO210mgQ2W:UST45mgQ12W | indrect | Low | Serious Imprecision |
| BRO210mgQ2W:UST90mgQ12W | indrect | Low | Serious Imprecision |
| GUS100mgQ4W:IXE80mgQ2W | indrect | Low | Serious Imprecision |
| GUS100mgQ4W:IXE80mgQ4W | indrect | Low | Serious Imprecision |
| GUS100mgQ4W:RIS150mg | indrect | Moderate | Heterogeneity |
| GUS100mgQ4W:SEC150mgQ4W | indrect | Low | Serious Imprecision |
| GUS100mgQ4W:SEC300mgQ4W | indrect | Low | Serious Imprecision |
| GUS100mgQ4W:TLD200mg | indrect | Low | Serious Imprecision |
| GUS100mgQ4W:UST45mgQ12W | indrect | Moderate | Imprecision |
| GUS100mgQ4W:UST90mgQ12W | indrect | Low | Serious Imprecision |
| GUS100mgQ8W:IXE80mgQ2W | indrect | Low | Serious Imprecision |
| GUS100mgQ8W:IXE80mgQ4W | indrect | Low | Serious Imprecision |
| GUS100mgQ8W:RIS150mg | indrect | Moderate | Imprecision |
| GUS100mgQ8W:SEC150mgQ4W | indrect | Low | Serious Imprecision |
| GUS100mgQ8W:SEC300mgQ4W | indrect | Moderate | Imprecision |
| GUS100mgQ8W:TLD200mg | indrect | Low | Serious Imprecision |
| GUS100mgQ8W:UST45mgQ12W | indrect | Moderate | Imprecision |
| GUS100mgQ8W:UST90mgQ12W | indrect | Low | Serious Imprecision |
| IXE80mgQ2W:RIS150mg | indrect | Moderate | Imprecision |
| IXE80mgQ2W:SEC150mgQ4W | indrect | Low | Serious Imprecision |
| IXE80mgQ2W:SEC300mgQ4W | indrect | Low | Serious Imprecision |
| IXE80mgQ2W:TLD200mg | indrect | Low | Serious Imprecision |
| IXE80mgQ2W:UST45mgQ12W | indrect | Low | Serious Imprecision |
| IXE80mgQ2W:UST90mgQ12W | indrect | Low | Serious Imprecision |
| IXE80mgQ4W:RIS150mg | indrect | Moderate | Imprecision |
| IXE80mgQ4W:SEC150mgQ4W | indrect | Low | Serious Imprecision |
| IXE80mgQ4W:SEC300mgQ4W | indrect | Low | Serious Imprecision |
| IXE80mgQ4W:TLD200mg | indrect | Low | Serious Imprecision |
| IXE80mgQ4W:UST45mgQ12W | indrect | Low | Serious Imprecision |
| IXE80mgQ4W:UST90mgQ12W | indrect | Low | Serious Imprecision |
| RIS150mg:SEC150mgQ4W | indrect | Moderate | Imprecision |
| RIS150mg:SEC300mgQ4W | indrect | High |  |
| RIS150mg:TLD200mg | indrect | Low | Serious Imprecision |
| RIS150mg:UST45mgQ12W | indrect | Low | Serious Imprecision |
| RIS150mg:UST90mgQ12W | indrect | Low | Imprecision、Heterogeneity |
| SEC150mgQ4W:TLD200mg | indrect | Low | Serious Imprecision |
| SEC150mgQ4W:UST45mgQ12W | indrect | Moderate | Imprecision |
| SEC150mgQ4W:UST90mgQ12W | indrect | Low | Serious Imprecision |
| SEC300mgQ4W:TLD200mg | indrect | Low | Serious Imprecision |
| SEC300mgQ4W:UST45mgQ12W | indrect | High |  |
| SEC300mgQ4W:UST90mgQ12W | indrect | Moderate | Imprecision |
| TLD200mg:UST45mgQ12W | indrect | Low | Serious Imprecision |
| TLD200mg:UST90mgQ12W | indrect | Low | Serious Imprecision |
| BKZ160mgQ4W:PLACEBO | mixed | High |  |
| BRO140mgQ2W:BRO210mgQ2W | mixed | Low | Imprecision、Heterogeneity |
| BRO140mgQ2W:PLACEBO | mixed | Low | Serious Incoherence |
| BRO210mgQ2W:PLACEBO | mixed | High |  |
| GUS100mgQ4W:GUS100mgQ8W | mixed | Low | Imprecision、Heterogeneity |
| GUS100mgQ4W:PLACEBO | mixed | High |  |
| GUS100mgQ8W:PLACEBO | mixed | High |  |
| IXE80mgQ2W:IXE80mgQ4W | mixed | Low | Serious Imprecision |
| IXE80mgQ2W:PLACEBO | mixed | High |  |
| IXE80mgQ4W:PLACEBO | mixed | High |  |
| PLACEBO:RIS150mg | mixed | High |  |
| PLACEBO:SEC150mgQ4W | mixed | High |  |
| PLACEBO:SEC300mgQ4W | mixed | High |  |
| PLACEBO:TLD200mg | mixed | High |  |
| PLACEBO:UST45mgQ12W | mixed | High |  |
| PLACEBO:UST90mgQ12W | mixed | High |  |
| SEC150mgQ4W:SEC300mgQ4W | mixed | Moderate | Imprecision |
| UST45mgQ12W:UST90mgQ12W | mixed | Moderate | Imprecision |
| BKZ160mgQ4W:BRO140mgQ2W | indrect | High |  |
| BKZ160mgQ4W:BRO210mgQ2W | indrect | High |  |
| BKZ160mgQ4W:GUS100mgQ4W | indrect | High |  |
| BKZ160mgQ4W:GUS100mgQ8W | indrect | High |  |
| BKZ160mgQ4W:IXE80mgQ2W | indrect | High |  |
| BKZ160mgQ4W:IXE80mgQ4W | indrect | High |  |
| BKZ160mgQ4W:RIS150mg | indrect | High |  |
| BKZ160mgQ4W:SEC150mgQ4W | indrect | High |  |
| BKZ160mgQ4W:SEC300mgQ4W | indrect | Moderate | Heterogeneity |
| BKZ160mgQ4W:TLD200mg | indrect | Low | Serious Imprecision |
| BKZ160mgQ4W:UST45mgQ12W | indrect | High |  |
| BKZ160mgQ4W:UST90mgQ12W | indrect | High |  |
| BRO140mgQ2W:GUS100mgQ4W | indrect | Low | Serious Imprecision |
| BRO140mgQ2W:GUS100mgQ8W | indrect | Low | Serious Imprecision |
| BRO140mgQ2W:IXE80mgQ2W | indrect | Low | Serious Imprecision |
| BRO140mgQ2W:IXE80mgQ4W | indrect | Low | Serious Imprecision |
| BRO140mgQ2W:RIS150mg | indrect | Low | Serious Imprecision |
| BRO140mgQ2W:SEC150mgQ4W | indrect | Low | Serious Imprecision |
| BRO140mgQ2W:SEC300mgQ4W | indrect | Moderate | Imprecision |
| BRO140mgQ2W:TLD200mg | indrect | Low | Serious Imprecision |
| BRO140mgQ2W:UST45mgQ12W | indrect | Low | Serious Imprecision |
| BRO140mgQ2W:UST90mgQ12W | indrect | Low | Serious Imprecision |
| BRO210mgQ2W:GUS100mgQ4W | indrect | Low | Serious Imprecision |
| BRO210mgQ2W:GUS100mgQ8W | indrect | Low | Serious Imprecision |
| BRO210mgQ2W:IXE80mgQ2W | indrect | Low | Serious Imprecision |
| BRO210mgQ2W:IXE80mgQ4W | indrect | Low | Serious Imprecision |
| BRO210mgQ2W:RIS150mg | indrect | Moderate | Imprecision |
| BRO210mgQ2W:SEC150mgQ4W | indrect | Low | Serious Imprecision |
| BRO210mgQ2W:SEC300mgQ4W | indrect | Low | Imprecision、Heterogeneity |
| BRO210mgQ2W:TLD200mg | indrect | Low | Serious Imprecision |
| BRO210mgQ2W:UST45mgQ12W | indrect | Low | Serious Imprecision |
| BRO210mgQ2W:UST90mgQ12W | indrect | Low | Serious Imprecision |
| GUS100mgQ4W:IXE80mgQ2W | indrect | Low | Serious Imprecision |
| GUS100mgQ4W:IXE80mgQ4W | indrect | Low | Serious Imprecision |
| GUS100mgQ4W:RIS150mg | indrect | Moderate | Heterogeneity |
| GUS100mgQ4W:SEC150mgQ4W | indrect | Low | Serious Imprecision |
| GUS100mgQ4W:SEC300mgQ4W | indrect | Low | Serious Imprecision |
| GUS100mgQ4W:TLD200mg | indrect | Low | Serious Imprecision |
| GUS100mgQ4W:UST45mgQ12W | indrect | Moderate | Imprecision |
| GUS100mgQ4W:UST90mgQ12W | indrect | Low | Serious Imprecision |
| GUS100mgQ8W:IXE80mgQ2W | indrect | Low | Serious Imprecision |
| GUS100mgQ8W:IXE80mgQ4W | indrect | Low | Serious Imprecision |
| GUS100mgQ8W:RIS150mg | indrect | Moderate | Imprecision |
| GUS100mgQ8W:SEC150mgQ4W | indrect | Low | Serious Imprecision |
| GUS100mgQ8W:SEC300mgQ4W | indrect | Moderate | Imprecision |
| GUS100mgQ8W:TLD200mg | indrect | Low | Serious Imprecision |
| GUS100mgQ8W:UST45mgQ12W | indrect | Moderate | Imprecision |
| GUS100mgQ8W:UST90mgQ12W | indrect | Low | Serious Imprecision |
| IXE80mgQ2W:RIS150mg | indrect | Moderate | Imprecision |
| IXE80mgQ2W:SEC150mgQ4W | indrect | Low | Serious Imprecision |
| IXE80mgQ2W:SEC300mgQ4W | indrect | Low | Serious Imprecision |
| IXE80mgQ2W:TLD200mg | indrect | Low | Serious Imprecision |
| IXE80mgQ2W:UST45mgQ12W | indrect | Low | Serious Imprecision |
| IXE80mgQ2W:UST90mgQ12W | indrect | Low | Serious Imprecision |
| IXE80mgQ4W:RIS150mg | indrect | Moderate | Imprecision |
| IXE80mgQ4W:SEC150mgQ4W | indrect | Low | Serious Imprecision |
| IXE80mgQ4W:SEC300mgQ4W | indrect | Low | Serious Imprecision |
| IXE80mgQ4W:TLD200mg | indrect | Low | Serious Imprecision |
| IXE80mgQ4W:UST45mgQ12W | indrect | Low | Serious Imprecision |
| IXE80mgQ4W:UST90mgQ12W | indrect | Low | Serious Imprecision |
| RIS150mg:SEC150mgQ4W | indrect | Moderate | Imprecision |
| RIS150mg:SEC300mgQ4W | indrect | High |  |
| RIS150mg:TLD200mg | indrect | Low | Serious Imprecision |
| RIS150mg:UST45mgQ12W | indrect | Low | Serious Imprecision |
| RIS150mg:UST90mgQ12W | indrect | Low | Imprecision、Heterogeneity |
| SEC150mgQ4W:TLD200mg | indrect | Low | Serious Imprecision |
| SEC150mgQ4W:UST45mgQ12W | indrect | Moderate | Imprecision |
| SEC150mgQ4W:UST90mgQ12W | indrect | Low | Serious Imprecision |
| SEC300mgQ4W:TLD200mg | indrect | Low | Serious Imprecision |
| SEC300mgQ4W:UST45mgQ12W | indrect | High |  |
| SEC300mgQ4W:UST90mgQ12W | indrect | Moderate | Imprecision |
| TLD200mg:UST45mgQ12W | indrect | Low | Serious Imprecision |
| TLD200mg:UST90mgQ12W | indrect | Low | Serious Imprecision |
| **ACR50** | | | |
| BKZ160mgQ4W:PLACEBO | mixed | High |  |
| BRO140mgQ2W:BRO210mgQ2W | mixed | Low | Serious Imprecision |
| BRO140mgQ2W:PLACEBO | mixed | Low | Serious Incoherence |
| BRO210mgQ2W:PLACEBO | mixed | High |  |
| GUS100mgQ4W:GUS100mgQ8W | mixed | Low | Serious Imprecision |
| GUS100mgQ4W:PLACEBO | mixed | High |  |
| GUS100mgQ8W:PLACEBO | mixed | High |  |
| IXE80mgQ2W:IXE80mgQ4W | mixed | Low | Serious Imprecision |
| IXE80mgQ2W:PLACEBO | mixed | High |  |
| IXE80mgQ4W:PLACEBO | mixed | High |  |
| PLACEBO:RIS150mg | mixed | High |  |
| PLACEBO:SEC150mgQ4W | mixed | High |  |
| PLACEBO:SEC300mgQ4W | mixed | High |  |
| PLACEBO:TLD200mg | mixed | High |  |
| PLACEBO:UST45mgQ12W | mixed | High |  |
| PLACEBO:UST90mgQ12W | mixed | High |  |
| SEC150mgQ4W:SEC300mgQ4W | mixed | Moderate | Imprecision |
| UST45mgQ12W:UST90mgQ12W | mixed | Low |  |
| BKZ160mgQ4W:BRO140mgQ2W | indrect | Moderate | Imprecision |
| BKZ160mgQ4W:BRO210mgQ2W | indrect | Low | Imprecision、Heterogeneity |
| BKZ160mgQ4W:GUS100mgQ4W | indrect | High |  |
| BKZ160mgQ4W:GUS100mgQ8W | indrect | High |  |
| BKZ160mgQ4W:IXE80mgQ2W | indrect | Low | Serious Imprecision |
| BKZ160mgQ4W:IXE80mgQ4W | indrect | Low | Serious Imprecision |
| BKZ160mgQ4W:RIS150mg | indrect | High |  |
| BKZ160mgQ4W:SEC150mgQ4W | indrect | Moderate | Imprecision |
| BKZ160mgQ4W:SEC300mgQ4W | indrect | Low | Imprecision、Heterogeneity |
| BKZ160mgQ4W:TLD200mg | indrect | Moderate | Heterogeneity |
| BKZ160mgQ4W:UST45mgQ12W | indrect | High |  |
| BKZ160mgQ4W:UST90mgQ12W | indrect | Moderate | Heterogeneity |
| BRO140mgQ2W:GUS100mgQ4W | indrect | Low | Serious Imprecision |
| BRO140mgQ2W:GUS100mgQ8W | indrect | Low | Serious Imprecision |
| BRO140mgQ2W:IXE80mgQ2W | indrect | Low | Serious Imprecision |
| BRO140mgQ2W:IXE80mgQ4W | indrect | Low | Serious Imprecision |
| BRO140mgQ2W:RIS150mg | indrect | Low | Serious Imprecision |
| BRO140mgQ2W:SEC150mgQ4W | indrect | Low | Serious Imprecision |
| BRO140mgQ2W:SEC300mgQ4W | indrect | Low | Serious Imprecision |
| BRO140mgQ2W:TLD200mg | indrect | Low | Serious Imprecision |
| BRO140mgQ2W:UST45mgQ12W | indrect | Low | Serious Imprecision |
| BRO140mgQ2W:UST90mgQ12W | indrect | Low | Serious Imprecision |
| BRO210mgQ2W:GUS100mgQ4W | indrect | Low | Serious Imprecision |
| BRO210mgQ2W:GUS100mgQ8W | indrect | Low | Serious Imprecision |
| BRO210mgQ2W:IXE80mgQ2W | indrect | Low | Serious Imprecision |
| BRO210mgQ2W:IXE80mgQ4W | indrect | Low | Serious Imprecision |
| BRO210mgQ2W:RIS150mg | indrect | Low | Serious Imprecision |
| BRO210mgQ2W:SEC150mgQ4W | indrect | Low | Serious Imprecision |
| BRO210mgQ2W:SEC300mgQ4W | indrect | Low | Serious Imprecision |
| BRO210mgQ2W:TLD200mg | indrect | Low | Serious Imprecision |
| BRO210mgQ2W:UST45mgQ12W | indrect | Low | Serious Imprecision |
| BRO210mgQ2W:UST90mgQ12W | indrect | Low | Serious Imprecision |
| GUS100mgQ4W:IXE80mgQ2W | indrect | Low | Serious Imprecision |
| GUS100mgQ4W:IXE80mgQ4W | indrect | Low | Serious Imprecision |
| GUS100mgQ4W:RIS150mg | indrect | Low | Serious Imprecision |
| GUS100mgQ4W:SEC150mgQ4W | indrect | Low | Serious Imprecision |
| GUS100mgQ4W:SEC300mgQ4W | indrect | Moderate | Imprecision |
| GUS100mgQ4W:TLD200mg | indrect | Low | Serious Imprecision |
| GUS100mgQ4W:UST45mgQ12W | indrect | Low | Serious Imprecision |
| GUS100mgQ4W:UST90mgQ12W | indrect | Low | Serious Imprecision |
| GUS100mgQ8W:IXE80mgQ2W | indrect | Low | Imprecision、Heterogeneity |
| GUS100mgQ8W:IXE80mgQ4W | indrect | Low | Imprecision、Heterogeneity |
| GUS100mgQ8W:RIS150mg | indrect | Low | Serious Imprecision |
| GUS100mgQ8W:SEC150mgQ4W | indrect | Low | Imprecision、Heterogeneity |
| GUS100mgQ8W:SEC300mgQ4W | indrect | High |  |
| GUS100mgQ8W:TLD200mg | indrect | Low | Serious Imprecision |
| GUS100mgQ8W:UST45mgQ12W | indrect | Low | Serious Imprecision |
| GUS100mgQ8W:UST90mgQ12W | indrect | Low | Serious Imprecision |
| IXE80mgQ2W:RIS150mg | indrect | Low | Serious Imprecision |
| IXE80mgQ2W:SEC150mgQ4W | indrect | Low | Serious Imprecision |
| IXE80mgQ2W:SEC300mgQ4W | indrect | Low | Serious Imprecision |
| IXE80mgQ2W:TLD200mg | indrect | Low | Serious Imprecision |
| IXE80mgQ2W:UST45mgQ12W | indrect | Low | Imprecision、Heterogeneity |
| IXE80mgQ2W:UST90mgQ12W | indrect | Low | Serious Imprecision |
| IXE80mgQ4W:RIS150mg | indrect | Low | Serious Imprecision |
| IXE80mgQ4W:SEC150mgQ4W | indrect | Low | Serious Imprecision |
| IXE80mgQ4W:SEC300mgQ4W | indrect | Low | Serious Imprecision |
| IXE80mgQ4W:TLD200mg | indrect | Low | Serious Imprecision |
| IXE80mgQ4W:UST45mgQ12W | indrect | Low | Serious Imprecision |
| IXE80mgQ4W:UST90mgQ12W | indrect | Low | Serious Imprecision |
| RIS150mg:SEC150mgQ4W | indrect | Low | Serious Imprecision |
| RIS150mg:SEC300mgQ4W | indrect | Moderate | Heterogeneity |
| RIS150mg:TLD200mg | indrect | Low | Serious Imprecision |
| RIS150mg:UST45mgQ12W | indrect | Low | Serious Imprecision |
| RIS150mg:UST90mgQ12W | indrect | Low | Serious Imprecision |
| SEC150mgQ4W:TLD200mg | indrect | Low | Serious Imprecision |
| SEC150mgQ4W:UST45mgQ12W | indrect | Low | Serious Imprecision |
| SEC150mgQ4W:UST90mgQ12W | indrect | Low | Serious Imprecision |
| SEC300mgQ4W:TLD200mg | indrect | Low | Imprecision、Heterogeneity |
| SEC300mgQ4W:UST45mgQ12W | indrect | Moderate | Heterogeneity |
| SEC300mgQ4W:UST90mgQ12W | indrect | Low | Imprecision、Heterogeneity |
| TLD200mg:UST45mgQ12W | indrect | Low | Serious Imprecision |
| TLD200mg:UST90mgQ12W | indrect | Low | Serious Imprecision |
| BKZ160mgQ4W:PLACEBO | mixed | High |  |
| BRO140mgQ2W:BRO210mgQ2W | mixed | Low | Serious Imprecision |
| BRO140mgQ2W:PLACEBO | mixed | Low | Serious Incoherence |
| BRO210mgQ2W:PLACEBO | mixed | High |  |
| GUS100mgQ4W:GUS100mgQ8W | mixed | Low | Serious Imprecision |
| GUS100mgQ4W:PLACEBO | mixed | High |  |
| GUS100mgQ8W:PLACEBO | mixed | High |  |
| IXE80mgQ2W:IXE80mgQ4W | mixed | Low | Serious Imprecision |
| IXE80mgQ2W:PLACEBO | mixed | High |  |
| IXE80mgQ4W:PLACEBO | mixed | High |  |
| PLACEBO:RIS150mg | mixed | High |  |
| PLACEBO:SEC150mgQ4W | mixed | High |  |
| PLACEBO:SEC300mgQ4W | mixed | High |  |
| PLACEBO:TLD200mg | mixed | High |  |
| PLACEBO:UST45mgQ12W | mixed | High |  |
| PLACEBO:UST90mgQ12W | mixed | High |  |
| SEC150mgQ4W:SEC300mgQ4W | mixed | Moderate | Imprecision |
| UST45mgQ12W:UST90mgQ12W | mixed | Low |  |
| BKZ160mgQ4W:BRO140mgQ2W | indrect | Moderate | Imprecision |
| BKZ160mgQ4W:BRO210mgQ2W | indrect | Low | Imprecision、Heterogeneity |
| BKZ160mgQ4W:GUS100mgQ4W | indrect | High |  |
| BKZ160mgQ4W:GUS100mgQ8W | indrect | High |  |
| BKZ160mgQ4W:IXE80mgQ2W | indrect | Low | Serious Imprecision |
| BKZ160mgQ4W:IXE80mgQ4W | indrect | Low | Serious Imprecision |
| BKZ160mgQ4W:RIS150mg | indrect | High |  |
| BKZ160mgQ4W:SEC150mgQ4W | indrect | Moderate | Imprecision |
| BKZ160mgQ4W:SEC300mgQ4W | indrect | Low | Imprecision、Heterogeneity |
| BKZ160mgQ4W:TLD200mg | indrect | Moderate | Heterogeneity |
| BKZ160mgQ4W:UST45mgQ12W | indrect | High |  |
| BKZ160mgQ4W:UST90mgQ12W | indrect | Moderate | Heterogeneity |
| BRO140mgQ2W:GUS100mgQ4W | indrect | Low | Serious Imprecision |
| BRO140mgQ2W:GUS100mgQ8W | indrect | Low | Serious Imprecision |
| BRO140mgQ2W:IXE80mgQ2W | indrect | Low | Serious Imprecision |
| BRO140mgQ2W:IXE80mgQ4W | indrect | Low | Serious Imprecision |
| BRO140mgQ2W:RIS150mg | indrect | Low | Serious Imprecision |
| BRO140mgQ2W:SEC150mgQ4W | indrect | Low | Serious Imprecision |
| BRO140mgQ2W:SEC300mgQ4W | indrect | Low | Serious Imprecision |
| BRO140mgQ2W:TLD200mg | indrect | Low | Serious Imprecision |
| BRO140mgQ2W:UST45mgQ12W | indrect | Low | Serious Imprecision |
| BRO140mgQ2W:UST90mgQ12W | indrect | Low | Serious Imprecision |
| BRO210mgQ2W:GUS100mgQ4W | indrect | Low | Serious Imprecision |
| BRO210mgQ2W:GUS100mgQ8W | indrect | Low | Serious Imprecision |
| BRO210mgQ2W:IXE80mgQ2W | indrect | Low | Serious Imprecision |
| BRO210mgQ2W:IXE80mgQ4W | indrect | Low | Serious Imprecision |
| BRO210mgQ2W:RIS150mg | indrect | Low | Serious Imprecision |
| BRO210mgQ2W:SEC150mgQ4W | indrect | Low | Serious Imprecision |
| BRO210mgQ2W:SEC300mgQ4W | indrect | Low | Serious Imprecision |
| BRO210mgQ2W:TLD200mg | indrect | Low | Serious Imprecision |
| BRO210mgQ2W:UST45mgQ12W | indrect | Low | Serious Imprecision |
| BRO210mgQ2W:UST90mgQ12W | indrect | Low | Serious Imprecision |
| GUS100mgQ4W:IXE80mgQ2W | indrect | Low | Serious Imprecision |
| GUS100mgQ4W:IXE80mgQ4W | indrect | Low | Serious Imprecision |
| GUS100mgQ4W:RIS150mg | indrect | Low | Serious Imprecision |
| GUS100mgQ4W:SEC150mgQ4W | indrect | Low | Serious Imprecision |
| GUS100mgQ4W:SEC300mgQ4W | indrect | Moderate | Imprecision |
| GUS100mgQ4W:TLD200mg | indrect | Low | Serious Imprecision |
| GUS100mgQ4W:UST45mgQ12W | indrect | Low | Serious Imprecision |
| GUS100mgQ4W:UST90mgQ12W | indrect | Low | Serious Imprecision |
| GUS100mgQ8W:IXE80mgQ2W | indrect | Low | Imprecision、Heterogeneity |
| GUS100mgQ8W:IXE80mgQ4W | indrect | Low | Imprecision、Heterogeneity |
| GUS100mgQ8W:RIS150mg | indrect | Low | Serious Imprecision |
| GUS100mgQ8W:SEC150mgQ4W | indrect | Low | Imprecision、Heterogeneity |
| GUS100mgQ8W:SEC300mgQ4W | indrect | High |  |
| GUS100mgQ8W:TLD200mg | indrect | Low | Serious Imprecision |
| GUS100mgQ8W:UST45mgQ12W | indrect | Low | Serious Imprecision |
| GUS100mgQ8W:UST90mgQ12W | indrect | Low | Serious Imprecision |
| IXE80mgQ2W:RIS150mg | indrect | Low | Serious Imprecision |
| IXE80mgQ2W:SEC150mgQ4W | indrect | Low | Serious Imprecision |
| IXE80mgQ2W:SEC300mgQ4W | indrect | Low | Serious Imprecision |
| IXE80mgQ2W:TLD200mg | indrect | Low | Serious Imprecision |
| IXE80mgQ2W:UST45mgQ12W | indrect | Low | Imprecision、Heterogeneity |
| IXE80mgQ2W:UST90mgQ12W | indrect | Low | Serious Imprecision |
| IXE80mgQ4W:RIS150mg | indrect | Low | Serious Imprecision |
| IXE80mgQ4W:SEC150mgQ4W | indrect | Low | Serious Imprecision |
| IXE80mgQ4W:SEC300mgQ4W | indrect | Low | Serious Imprecision |
| IXE80mgQ4W:TLD200mg | indrect | Low | Serious Imprecision |
| IXE80mgQ4W:UST45mgQ12W | indrect | Low | Serious Imprecision |
| IXE80mgQ4W:UST90mgQ12W | indrect | Low | Serious Imprecision |
| RIS150mg:SEC150mgQ4W | indrect | Low | Serious Imprecision |
| RIS150mg:SEC300mgQ4W | indrect | Moderate | Heterogeneity |
| RIS150mg:TLD200mg | indrect | Low | Serious Imprecision |
| RIS150mg:UST45mgQ12W | indrect | Low | Serious Imprecision |
| RIS150mg:UST90mgQ12W | indrect | Low | Serious Imprecision |
| SEC150mgQ4W:TLD200mg | indrect | Low | Serious Imprecision |
| SEC150mgQ4W:UST45mgQ12W | indrect | Low | Serious Imprecision |
| SEC150mgQ4W:UST90mgQ12W | indrect | Low | Serious Imprecision |
| SEC300mgQ4W:TLD200mg | indrect | Low | Imprecision、Heterogeneity |
| SEC300mgQ4W:UST45mgQ12W | indrect | Moderate | Heterogeneity |
| SEC300mgQ4W:UST90mgQ12W | indrect | Low | Imprecision、Heterogeneity |
| TLD200mg:UST45mgQ12W | indrect | Low | Serious Imprecision |
| TLD200mg:UST90mgQ12W | indrect | Low | Serious Imprecision |
| BKZ160mgQ4W:PLACEBO | mixed | High |  |
| BRO140mgQ2W:BRO210mgQ2W | mixed | Low | Serious Imprecision |
| BRO140mgQ2W:PLACEBO | mixed | Low | Serious Incoherence |
| BRO210mgQ2W:PLACEBO | mixed | High |  |
| GUS100mgQ4W:GUS100mgQ8W | mixed | Low | Serious Imprecision |
| GUS100mgQ4W:PLACEBO | mixed | High |  |
| GUS100mgQ8W:PLACEBO | mixed | High |  |
| IXE80mgQ2W:IXE80mgQ4W | mixed | Low | Serious Imprecision |
| IXE80mgQ2W:PLACEBO | mixed | High |  |
| IXE80mgQ4W:PLACEBO | mixed | High |  |
| PLACEBO:RIS150mg | mixed | High |  |
| PLACEBO:SEC150mgQ4W | mixed | High |  |
| PLACEBO:SEC300mgQ4W | mixed | High |  |
| PLACEBO:TLD200mg | mixed | High |  |
| PLACEBO:UST45mgQ12W | mixed | High |  |
| PLACEBO:UST90mgQ12W | mixed | High |  |
| SEC150mgQ4W:SEC300mgQ4W | mixed | Moderate | Imprecision |
| UST45mgQ12W:UST90mgQ12W | mixed | Low |  |
| BKZ160mgQ4W:BRO140mgQ2W | indrect | Moderate | Imprecision |
| BKZ160mgQ4W:BRO210mgQ2W | indrect | Low | Imprecision、Heterogeneity |
| BKZ160mgQ4W:GUS100mgQ4W | indrect | High |  |
| BKZ160mgQ4W:GUS100mgQ8W | indrect | High |  |
| BKZ160mgQ4W:IXE80mgQ2W | indrect | Low | Serious Imprecision |
| BKZ160mgQ4W:IXE80mgQ4W | indrect | Low | Serious Imprecision |
| BKZ160mgQ4W:RIS150mg | indrect | High |  |
| BKZ160mgQ4W:SEC150mgQ4W | indrect | Moderate | Imprecision |
| BKZ160mgQ4W:SEC300mgQ4W | indrect | Low | Imprecision、Heterogeneity |
| BKZ160mgQ4W:TLD200mg | indrect | Moderate | Heterogeneity |
| BKZ160mgQ4W:UST45mgQ12W | indrect | High |  |
| BKZ160mgQ4W:UST90mgQ12W | indrect | Moderate | Heterogeneity |
| BRO140mgQ2W:GUS100mgQ4W | indrect | Low | Serious Imprecision |
| BRO140mgQ2W:GUS100mgQ8W | indrect | Low | Serious Imprecision |
| BRO140mgQ2W:IXE80mgQ2W | indrect | Low | Serious Imprecision |
| BRO140mgQ2W:IXE80mgQ4W | indrect | Low | Serious Imprecision |
| BRO140mgQ2W:RIS150mg | indrect | Low | Serious Imprecision |
| BRO140mgQ2W:SEC150mgQ4W | indrect | Low | Serious Imprecision |
| BRO140mgQ2W:SEC300mgQ4W | indrect | Low | Serious Imprecision |
| BRO140mgQ2W:TLD200mg | indrect | Low | Serious Imprecision |
| BRO140mgQ2W:UST45mgQ12W | indrect | Low | Serious Imprecision |
| BRO140mgQ2W:UST90mgQ12W | indrect | Low | Serious Imprecision |
| BRO210mgQ2W:GUS100mgQ4W | indrect | Low | Serious Imprecision |
| BRO210mgQ2W:GUS100mgQ8W | indrect | Low | Serious Imprecision |
| BRO210mgQ2W:IXE80mgQ2W | indrect | Low | Serious Imprecision |
| BRO210mgQ2W:IXE80mgQ4W | indrect | Low | Serious Imprecision |
| BRO210mgQ2W:RIS150mg | indrect | Low | Serious Imprecision |
| BRO210mgQ2W:SEC150mgQ4W | indrect | Low | Serious Imprecision |
| BRO210mgQ2W:SEC300mgQ4W | indrect | Low | Serious Imprecision |
| BRO210mgQ2W:TLD200mg | indrect | Low | Serious Imprecision |
| BRO210mgQ2W:UST45mgQ12W | indrect | Low | Serious Imprecision |
| BRO210mgQ2W:UST90mgQ12W | indrect | Low | Serious Imprecision |
| GUS100mgQ4W:IXE80mgQ2W | indrect | Low | Serious Imprecision |
| GUS100mgQ4W:IXE80mgQ4W | indrect | Low | Serious Imprecision |
| GUS100mgQ4W:RIS150mg | indrect | Low | Serious Imprecision |
| GUS100mgQ4W:SEC150mgQ4W | indrect | Low | Serious Imprecision |
| GUS100mgQ4W:SEC300mgQ4W | indrect | Moderate | Imprecision |
| GUS100mgQ4W:TLD200mg | indrect | Low | Serious Imprecision |
| GUS100mgQ4W:UST45mgQ12W | indrect | Low | Serious Imprecision |
| GUS100mgQ4W:UST90mgQ12W | indrect | Low | Serious Imprecision |
| GUS100mgQ8W:IXE80mgQ2W | indrect | Low | Imprecision、Heterogeneity |
| GUS100mgQ8W:IXE80mgQ4W | indrect | Low | Imprecision、Heterogeneity |
| GUS100mgQ8W:RIS150mg | indrect | Low | Serious Imprecision |
| GUS100mgQ8W:SEC150mgQ4W | indrect | Low | Imprecision、Heterogeneity |
| GUS100mgQ8W:SEC300mgQ4W | indrect | High |  |
| GUS100mgQ8W:TLD200mg | indrect | Low | Serious Imprecision |
| GUS100mgQ8W:UST45mgQ12W | indrect | Low | Serious Imprecision |
| GUS100mgQ8W:UST90mgQ12W | indrect | Low | Serious Imprecision |
| IXE80mgQ2W:RIS150mg | indrect | Low | Serious Imprecision |
| IXE80mgQ2W:SEC150mgQ4W | indrect | Low | Serious Imprecision |
| IXE80mgQ2W:SEC300mgQ4W | indrect | Low | Serious Imprecision |
| IXE80mgQ2W:TLD200mg | indrect | Low | Serious Imprecision |
| IXE80mgQ2W:UST45mgQ12W | indrect | Low | Imprecision、Heterogeneity |
| IXE80mgQ2W:UST90mgQ12W | indrect | Low | Serious Imprecision |
| IXE80mgQ4W:RIS150mg | indrect | Low | Serious Imprecision |
| IXE80mgQ4W:SEC150mgQ4W | indrect | Low | Serious Imprecision |
| IXE80mgQ4W:SEC300mgQ4W | indrect | Low | Serious Imprecision |
| IXE80mgQ4W:TLD200mg | indrect | Low | Serious Imprecision |
| IXE80mgQ4W:UST45mgQ12W | indrect | Low | Serious Imprecision |
| IXE80mgQ4W:UST90mgQ12W | indrect | Low | Serious Imprecision |
| RIS150mg:SEC150mgQ4W | indrect | Low | Serious Imprecision |
| RIS150mg:SEC300mgQ4W | indrect | Moderate | Heterogeneity |
| RIS150mg:TLD200mg | indrect | Low | Serious Imprecision |
| RIS150mg:UST45mgQ12W | indrect | Low | Serious Imprecision |
| RIS150mg:UST90mgQ12W | indrect | Low | Serious Imprecision |
| SEC150mgQ4W:TLD200mg | indrect | Low | Serious Imprecision |
| SEC150mgQ4W:UST45mgQ12W | indrect | Low | Serious Imprecision |
| SEC150mgQ4W:UST90mgQ12W | indrect | Low | Serious Imprecision |
| SEC300mgQ4W:TLD200mg | indrect | Low | Imprecision、Heterogeneity |
| SEC300mgQ4W:UST45mgQ12W | indrect | Moderate | Heterogeneity |
| SEC300mgQ4W:UST90mgQ12W | indrect | Low | Imprecision、Heterogeneity |
| TLD200mg:UST45mgQ12W | indrect | Low | Serious Imprecision |
| TLD200mg:UST90mgQ12W | indrect | Low | Serious Imprecision |
| **ACR70** | | | |
| BKZ160mgQ4W:PLACEBO | mixed | High |  |
| BRO140mgQ2W:BRO210mgQ2W | mixed | Low | Serious Imprecision |
| BRO140mgQ2W:PLACEBO | mixed | High |  |
| BRO210mgQ2W:PLACEBO | mixed | High |  |
| GUS100mgQ4W:GUS100mgQ8W | mixed | Low | Serious Imprecision |
| GUS100mgQ4W:PLACEBO | mixed | High |  |
| GUS100mgQ8W:PLACEBO | mixed | High |  |
| IXE80mgQ2W:IXE80mgQ4W | mixed | Low | Serious Imprecision |
| IXE80mgQ2W:PLACEBO | mixed | Low | Serious Incoherence |
| IXE80mgQ4W:PLACEBO | mixed | High |  |
| PLACEBO:RIS150mg | mixed | High |  |
| PLACEBO:SEC150mgQ4W | mixed | High |  |
| PLACEBO:SEC300mgQ4W | mixed | High |  |
| PLACEBO:TLD200mg | mixed | Moderate | Heterogeneity |
| PLACEBO:UST45mgQ12W | mixed | High |  |
| PLACEBO:UST90mgQ12W | mixed | High |  |
| SEC150mgQ4W:SEC300mgQ4W | mixed | Low | Serious Imprecision |
| UST45mgQ12W:UST90mgQ12W | mixed | Low | Serious Imprecision |
| BKZ160mgQ4W:BRO140mgQ2W | indrect | Low | Serious Imprecision |
| BKZ160mgQ4W:BRO210mgQ2W | indrect | Low | Serious Imprecision |
| BKZ160mgQ4W:GUS100mgQ4W | indrect | Low | Serious Imprecision |
| BKZ160mgQ4W:GUS100mgQ8W | indrect | Low | Serious Imprecision |
| BKZ160mgQ4W:IXE80mgQ2W | indrect | Low | Serious Imprecision |
| BKZ160mgQ4W:IXE80mgQ4W | indrect | Low | Serious Imprecision |
| BKZ160mgQ4W:RIS150mg | indrect | Low | Serious Heterogeneity |
| BKZ160mgQ4W:SEC150mgQ4W | indrect | Low | Serious Imprecision |
| BKZ160mgQ4W:SEC300mgQ4W | indrect | Low | Serious Imprecision |
| BKZ160mgQ4W:TLD200mg | indrect | Low | Serious Imprecision |
| BKZ160mgQ4W:UST45mgQ12W | indrect | Low | Imprecision、Heterogeneity |
| BKZ160mgQ4W:UST90mgQ12W | indrect | Low | Serious Imprecision |
| BRO140mgQ2W:GUS100mgQ4W | indrect | Low | Serious Imprecision |
| BRO140mgQ2W:GUS100mgQ8W | indrect | Low | Serious Imprecision |
| BRO140mgQ2W:IXE80mgQ2W | indrect | Low | Serious Imprecision |
| BRO140mgQ2W:IXE80mgQ4W | indrect | Low | Serious Imprecision |
| BRO140mgQ2W:RIS150mg | indrect | Low | Serious Imprecision |
| BRO140mgQ2W:SEC150mgQ4W | indrect | Low | Serious Imprecision |
| BRO140mgQ2W:SEC300mgQ4W | indrect | Low | Serious Imprecision |
| BRO140mgQ2W:TLD200mg | indrect | Low | Serious Imprecision |
| BRO140mgQ2W:UST45mgQ12W | indrect | Low | Serious Imprecision |
| BRO140mgQ2W:UST90mgQ12W | indrect | Low | Serious Imprecision |
| BRO210mgQ2W:GUS100mgQ4W | indrect | Low | Serious Imprecision |
| BRO210mgQ2W:GUS100mgQ8W | indrect | Low | Serious Imprecision |
| BRO210mgQ2W:IXE80mgQ2W | indrect | Low | Serious Imprecision |
| BRO210mgQ2W:IXE80mgQ4W | indrect | Low | Serious Imprecision |
| BRO210mgQ2W:RIS150mg | indrect | Low | Serious Imprecision |
| BRO210mgQ2W:SEC150mgQ4W | indrect | Low | Serious Imprecision |
| BRO210mgQ2W:SEC300mgQ4W | indrect | Low | Serious Imprecision |
| BRO210mgQ2W:TLD200mg | indrect | Low | Serious Imprecision |
| BRO210mgQ2W:UST45mgQ12W | indrect | Low | Serious Imprecision |
| BRO210mgQ2W:UST90mgQ12W | indrect | Low | Serious Imprecision |
| GUS100mgQ4W:IXE80mgQ2W | indrect | Low | Serious Imprecision |
| GUS100mgQ4W:IXE80mgQ4W | indrect | Low | Serious Imprecision |
| GUS100mgQ4W:RIS150mg | indrect | Low | Serious Imprecision |
| GUS100mgQ4W:SEC150mgQ4W | indrect | Low | Serious Imprecision |
| GUS100mgQ4W:SEC300mgQ4W | indrect | Low | Serious Imprecision |
| GUS100mgQ4W:TLD200mg | indrect | Low | Serious Imprecision |
| GUS100mgQ4W:UST45mgQ12W | indrect | Low | Serious Imprecision |
| GUS100mgQ4W:UST90mgQ12W | indrect | Low | Serious Imprecision |
| GUS100mgQ8W:IXE80mgQ2W | indrect | Low | Serious Imprecision |
| GUS100mgQ8W:IXE80mgQ4W | indrect | Low | Serious Imprecision |
| GUS100mgQ8W:RIS150mg | indrect | Low | Serious Imprecision |
| GUS100mgQ8W:SEC150mgQ4W | indrect | Low | Serious Imprecision |
| GUS100mgQ8W:SEC300mgQ4W | indrect | Low | Serious Imprecision |
| GUS100mgQ8W:TLD200mg | indrect | Low | Serious Imprecision |
| GUS100mgQ8W:UST45mgQ12W | indrect | Low | Serious Imprecision |
| GUS100mgQ8W:UST90mgQ12W | indrect | Low | Serious Imprecision |
| IXE80mgQ2W:RIS150mg | indrect | Low | Imprecision、Heterogeneity |
| IXE80mgQ2W:SEC150mgQ4W | indrect | Low | Serious Imprecision |
| IXE80mgQ2W:SEC300mgQ4W | indrect | Low | Serious Imprecision |
| IXE80mgQ2W:TLD200mg | indrect | Low | Serious Imprecision |
| IXE80mgQ2W:UST45mgQ12W | indrect | Low | Serious Imprecision |
| IXE80mgQ2W:UST90mgQ12W | indrect | Low | Serious Imprecision |
| IXE80mgQ4W:RIS150mg | indrect | Low | Imprecision、Heterogeneity |
| IXE80mgQ4W:SEC150mgQ4W | indrect | Low | Serious Imprecision |
| IXE80mgQ4W:SEC300mgQ4W | indrect | Low | Serious Imprecision |
| IXE80mgQ4W:TLD200mg | indrect | Low | Serious Imprecision |
| IXE80mgQ4W:UST45mgQ12W | indrect | Low | Serious Imprecision |
| IXE80mgQ4W:UST90mgQ12W | indrect | Low | Serious Imprecision |
| RIS150mg:SEC150mgQ4W | indrect | Low | Imprecision、Heterogeneity |
| RIS150mg:SEC300mgQ4W | indrect | Low | Imprecision、Heterogeneity |
| RIS150mg:TLD200mg | indrect | Low | Serious Imprecision |
| RIS150mg:UST45mgQ12W | indrect | Low | Serious Imprecision |
| RIS150mg:UST90mgQ12W | indrect | Low | Serious Imprecision |
| SEC150mgQ4W:TLD200mg | indrect | Low | Serious Imprecision |
| SEC150mgQ4W:UST45mgQ12W | indrect | Low | Serious Imprecision |
| SEC150mgQ4W:UST90mgQ12W | indrect | Low | Serious Imprecision |
| SEC300mgQ4W:TLD200mg | indrect | Low | Serious Imprecision |
| SEC300mgQ4W:UST45mgQ12W | indrect | Low | Serious Imprecision |
| SEC300mgQ4W:UST90mgQ12W | indrect | Low | Serious Imprecision |
| TLD200mg:UST45mgQ12W | indrect | Low | Serious Imprecision |
| TLD200mg:UST90mgQ12W | indrect | Low | Serious Imprecision |
| BKZ160mgQ4W:PLACEBO | mixed | High |  |
| BRO140mgQ2W:BRO210mgQ2W | mixed | Low | Serious Imprecision |
| BRO140mgQ2W:PLACEBO | mixed | High |  |
| BRO210mgQ2W:PLACEBO | mixed | High |  |
| GUS100mgQ4W:GUS100mgQ8W | mixed | Low | Serious Imprecision |
| GUS100mgQ4W:PLACEBO | mixed | High |  |
| GUS100mgQ8W:PLACEBO | mixed | High |  |
| IXE80mgQ2W:IXE80mgQ4W | mixed | Low | Serious Imprecision |
| IXE80mgQ2W:PLACEBO | mixed | Low | Serious Incoherence |
| IXE80mgQ4W:PLACEBO | mixed | High |  |
| PLACEBO:RIS150mg | mixed | High |  |
| PLACEBO:SEC150mgQ4W | mixed | High |  |
| PLACEBO:SEC300mgQ4W | mixed | High |  |
| PLACEBO:TLD200mg | mixed | Moderate | Heterogeneity |
| PLACEBO:UST45mgQ12W | mixed | High |  |
| PLACEBO:UST90mgQ12W | mixed | High |  |
| SEC150mgQ4W:SEC300mgQ4W | mixed | Low | Serious Imprecision |
| UST45mgQ12W:UST90mgQ12W | mixed | Low | Serious Imprecision |
| BKZ160mgQ4W:BRO140mgQ2W | indrect | Low | Serious Imprecision |
| BKZ160mgQ4W:BRO210mgQ2W | indrect | Low | Serious Imprecision |
| BKZ160mgQ4W:GUS100mgQ4W | indrect | Low | Serious Imprecision |
| BKZ160mgQ4W:GUS100mgQ8W | indrect | Low | Serious Imprecision |
| BKZ160mgQ4W:IXE80mgQ2W | indrect | Low | Serious Imprecision |
| BKZ160mgQ4W:IXE80mgQ4W | indrect | Low | Serious Imprecision |
| BKZ160mgQ4W:RIS150mg | indrect | Low | Serious Heterogeneity |
| BKZ160mgQ4W:SEC150mgQ4W | indrect | Low | Serious Imprecision |
| BKZ160mgQ4W:SEC300mgQ4W | indrect | Low | Serious Imprecision |
| BKZ160mgQ4W:TLD200mg | indrect | Low | Serious Imprecision |
| BKZ160mgQ4W:UST45mgQ12W | indrect | Low | Imprecision、Heterogeneity |
| BKZ160mgQ4W:UST90mgQ12W | indrect | Low | Serious Imprecision |
| BRO140mgQ2W:GUS100mgQ4W | indrect | Low | Serious Imprecision |
| BRO140mgQ2W:GUS100mgQ8W | indrect | Low | Serious Imprecision |
| BRO140mgQ2W:IXE80mgQ2W | indrect | Low | Serious Imprecision |
| BRO140mgQ2W:IXE80mgQ4W | indrect | Low | Serious Imprecision |
| BRO140mgQ2W:RIS150mg | indrect | Low | Serious Imprecision |
| BRO140mgQ2W:SEC150mgQ4W | indrect | Low | Serious Imprecision |
| BRO140mgQ2W:SEC300mgQ4W | indrect | Low | Serious Imprecision |
| BRO140mgQ2W:TLD200mg | indrect | Low | Serious Imprecision |
| BRO140mgQ2W:UST45mgQ12W | indrect | Low | Serious Imprecision |
| BRO140mgQ2W:UST90mgQ12W | indrect | Low | Serious Imprecision |
| BRO210mgQ2W:GUS100mgQ4W | indrect | Low | Serious Imprecision |
| BRO210mgQ2W:GUS100mgQ8W | indrect | Low | Serious Imprecision |
| BRO210mgQ2W:IXE80mgQ2W | indrect | Low | Serious Imprecision |
| BRO210mgQ2W:IXE80mgQ4W | indrect | Low | Serious Imprecision |
| BRO210mgQ2W:RIS150mg | indrect | Low | Serious Imprecision |
| BRO210mgQ2W:SEC150mgQ4W | indrect | Low | Serious Imprecision |
| BRO210mgQ2W:SEC300mgQ4W | indrect | Low | Serious Imprecision |
| BRO210mgQ2W:TLD200mg | indrect | Low | Serious Imprecision |
| BRO210mgQ2W:UST45mgQ12W | indrect | Low | Serious Imprecision |
| BRO210mgQ2W:UST90mgQ12W | indrect | Low | Serious Imprecision |
| GUS100mgQ4W:IXE80mgQ2W | indrect | Low | Serious Imprecision |
| GUS100mgQ4W:IXE80mgQ4W | indrect | Low | Serious Imprecision |
| GUS100mgQ4W:RIS150mg | indrect | Low | Serious Imprecision |
| GUS100mgQ4W:SEC150mgQ4W | indrect | Low | Serious Imprecision |
| GUS100mgQ4W:SEC300mgQ4W | indrect | Low | Serious Imprecision |
| GUS100mgQ4W:TLD200mg | indrect | Low | Serious Imprecision |
| GUS100mgQ4W:UST45mgQ12W | indrect | Low | Serious Imprecision |
| GUS100mgQ4W:UST90mgQ12W | indrect | Low | Serious Imprecision |
| GUS100mgQ8W:IXE80mgQ2W | indrect | Low | Serious Imprecision |
| GUS100mgQ8W:IXE80mgQ4W | indrect | Low | Serious Imprecision |
| GUS100mgQ8W:RIS150mg | indrect | Low | Serious Imprecision |
| GUS100mgQ8W:SEC150mgQ4W | indrect | Low | Serious Imprecision |
| GUS100mgQ8W:SEC300mgQ4W | indrect | Low | Serious Imprecision |
| GUS100mgQ8W:TLD200mg | indrect | Low | Serious Imprecision |
| GUS100mgQ8W:UST45mgQ12W | indrect | Low | Serious Imprecision |
| GUS100mgQ8W:UST90mgQ12W | indrect | Low | Serious Imprecision |
| IXE80mgQ2W:RIS150mg | indrect | Low | Imprecision、Heterogeneity |
| IXE80mgQ2W:SEC150mgQ4W | indrect | Low | Serious Imprecision |
| IXE80mgQ2W:SEC300mgQ4W | indrect | Low | Serious Imprecision |
| IXE80mgQ2W:TLD200mg | indrect | Low | Serious Imprecision |
| IXE80mgQ2W:UST45mgQ12W | indrect | Low | Serious Imprecision |
| IXE80mgQ2W:UST90mgQ12W | indrect | Low | Serious Imprecision |
| IXE80mgQ4W:RIS150mg | indrect | Low | Imprecision、Heterogeneity |
| IXE80mgQ4W:SEC150mgQ4W | indrect | Low | Serious Imprecision |
| IXE80mgQ4W:SEC300mgQ4W | indrect | Low | Serious Imprecision |
| IXE80mgQ4W:TLD200mg | indrect | Low | Serious Imprecision |
| IXE80mgQ4W:UST45mgQ12W | indrect | Low | Serious Imprecision |
| IXE80mgQ4W:UST90mgQ12W | indrect | Low | Serious Imprecision |
| RIS150mg:SEC150mgQ4W | indrect | Low | Imprecision、Heterogeneity |
| RIS150mg:SEC300mgQ4W | indrect | Low | Imprecision、Heterogeneity |
| RIS150mg:TLD200mg | indrect | Low | Serious Imprecision |
| RIS150mg:UST45mgQ12W | indrect | Low | Serious Imprecision |
| RIS150mg:UST90mgQ12W | indrect | Low | Serious Imprecision |
| SEC150mgQ4W:TLD200mg | indrect | Low | Serious Imprecision |
| SEC150mgQ4W:UST45mgQ12W | indrect | Low | Serious Imprecision |
| SEC150mgQ4W:UST90mgQ12W | indrect | Low | Serious Imprecision |
| SEC300mgQ4W:TLD200mg | indrect | Low | Serious Imprecision |
| SEC300mgQ4W:UST45mgQ12W | indrect | Low | Serious Imprecision |
| SEC300mgQ4W:UST90mgQ12W | indrect | Low | Serious Imprecision |
| TLD200mg:UST45mgQ12W | indrect | Low | Serious Imprecision |
| TLD200mg:UST90mgQ12W | indrect | Low | Serious Imprecision |
| BKZ160mgQ4W:PLACEBO | mixed | High |  |
| BRO140mgQ2W:BRO210mgQ2W | mixed | Low | Serious Imprecision |
| BRO140mgQ2W:PLACEBO | mixed | High |  |
| BRO210mgQ2W:PLACEBO | mixed | High |  |
| GUS100mgQ4W:GUS100mgQ8W | mixed | Low | Serious Imprecision |
| GUS100mgQ4W:PLACEBO | mixed | High |  |
| GUS100mgQ8W:PLACEBO | mixed | High |  |
| IXE80mgQ2W:IXE80mgQ4W | mixed | Low | Serious Imprecision |
| IXE80mgQ2W:PLACEBO | mixed | Low | Serious Incoherence |
| IXE80mgQ4W:PLACEBO | mixed | High |  |
| PLACEBO:RIS150mg | mixed | High |  |
| PLACEBO:SEC150mgQ4W | mixed | High |  |
| PLACEBO:SEC300mgQ4W | mixed | High |  |
| PLACEBO:TLD200mg | mixed | Moderate | Heterogeneity |
| PLACEBO:UST45mgQ12W | mixed | High |  |
| PLACEBO:UST90mgQ12W | mixed | High |  |
| SEC150mgQ4W:SEC300mgQ4W | mixed | Low | Serious Imprecision |
| UST45mgQ12W:UST90mgQ12W | mixed | Low | Serious Imprecision |
| BKZ160mgQ4W:BRO140mgQ2W | indrect | Low | Serious Imprecision |
| BKZ160mgQ4W:BRO210mgQ2W | indrect | Low | Serious Imprecision |
| BKZ160mgQ4W:GUS100mgQ4W | indrect | Low | Serious Imprecision |
| BKZ160mgQ4W:GUS100mgQ8W | indrect | Low | Serious Imprecision |
| BKZ160mgQ4W:IXE80mgQ2W | indrect | Low | Serious Imprecision |
| BKZ160mgQ4W:IXE80mgQ4W | indrect | Low | Serious Imprecision |
| BKZ160mgQ4W:RIS150mg | indrect | Low | Serious Heterogeneity |
| BKZ160mgQ4W:SEC150mgQ4W | indrect | Low | Serious Imprecision |
| BKZ160mgQ4W:SEC300mgQ4W | indrect | Low | Serious Imprecision |
| BKZ160mgQ4W:TLD200mg | indrect | Low | Serious Imprecision |
| BKZ160mgQ4W:UST45mgQ12W | indrect | Low | Imprecision、Heterogeneity |
| BKZ160mgQ4W:UST90mgQ12W | indrect | Low | Serious Imprecision |
| BRO140mgQ2W:GUS100mgQ4W | indrect | Low | Serious Imprecision |
| BRO140mgQ2W:GUS100mgQ8W | indrect | Low | Serious Imprecision |
| BRO140mgQ2W:IXE80mgQ2W | indrect | Low | Serious Imprecision |
| BRO140mgQ2W:IXE80mgQ4W | indrect | Low | Serious Imprecision |
| BRO140mgQ2W:RIS150mg | indrect | Low | Serious Imprecision |
| BRO140mgQ2W:SEC150mgQ4W | indrect | Low | Serious Imprecision |
| BRO140mgQ2W:SEC300mgQ4W | indrect | Low | Serious Imprecision |
| BRO140mgQ2W:TLD200mg | indrect | Low | Serious Imprecision |
| BRO140mgQ2W:UST45mgQ12W | indrect | Low | Serious Imprecision |
| BRO140mgQ2W:UST90mgQ12W | indrect | Low | Serious Imprecision |
| BRO210mgQ2W:GUS100mgQ4W | indrect | Low | Serious Imprecision |
| BRO210mgQ2W:GUS100mgQ8W | indrect | Low | Serious Imprecision |
| BRO210mgQ2W:IXE80mgQ2W | indrect | Low | Serious Imprecision |
| BRO210mgQ2W:IXE80mgQ4W | indrect | Low | Serious Imprecision |
| BRO210mgQ2W:RIS150mg | indrect | Low | Serious Imprecision |
| BRO210mgQ2W:SEC150mgQ4W | indrect | Low | Serious Imprecision |
| BRO210mgQ2W:SEC300mgQ4W | indrect | Low | Serious Imprecision |
| BRO210mgQ2W:TLD200mg | indrect | Low | Serious Imprecision |
| BRO210mgQ2W:UST45mgQ12W | indrect | Low | Serious Imprecision |
| BRO210mgQ2W:UST90mgQ12W | indrect | Low | Serious Imprecision |
| GUS100mgQ4W:IXE80mgQ2W | indrect | Low | Serious Imprecision |
| GUS100mgQ4W:IXE80mgQ4W | indrect | Low | Serious Imprecision |
| GUS100mgQ4W:RIS150mg | indrect | Low | Serious Imprecision |
| GUS100mgQ4W:SEC150mgQ4W | indrect | Low | Serious Imprecision |
| GUS100mgQ4W:SEC300mgQ4W | indrect | Low | Serious Imprecision |
| GUS100mgQ4W:TLD200mg | indrect | Low | Serious Imprecision |
| GUS100mgQ4W:UST45mgQ12W | indrect | Low | Serious Imprecision |
| GUS100mgQ4W:UST90mgQ12W | indrect | Low | Serious Imprecision |
| GUS100mgQ8W:IXE80mgQ2W | indrect | Low | Serious Imprecision |
| GUS100mgQ8W:IXE80mgQ4W | indrect | Low | Serious Imprecision |
| GUS100mgQ8W:RIS150mg | indrect | Low | Serious Imprecision |
| GUS100mgQ8W:SEC150mgQ4W | indrect | Low | Serious Imprecision |
| GUS100mgQ8W:SEC300mgQ4W | indrect | Low | Serious Imprecision |
| GUS100mgQ8W:TLD200mg | indrect | Low | Serious Imprecision |
| GUS100mgQ8W:UST45mgQ12W | indrect | Low | Serious Imprecision |
| GUS100mgQ8W:UST90mgQ12W | indrect | Low | Serious Imprecision |
| IXE80mgQ2W:RIS150mg | indrect | Low | Imprecision、Heterogeneity |
| IXE80mgQ2W:SEC150mgQ4W | indrect | Low | Serious Imprecision |
| IXE80mgQ2W:SEC300mgQ4W | indrect | Low | Serious Imprecision |
| IXE80mgQ2W:TLD200mg | indrect | Low | Serious Imprecision |
| IXE80mgQ2W:UST45mgQ12W | indrect | Low | Serious Imprecision |
| IXE80mgQ2W:UST90mgQ12W | indrect | Low | Serious Imprecision |
| IXE80mgQ4W:RIS150mg | indrect | Low | Imprecision、Heterogeneity |
| IXE80mgQ4W:SEC150mgQ4W | indrect | Low | Serious Imprecision |
| IXE80mgQ4W:SEC300mgQ4W | indrect | Low | Serious Imprecision |
| IXE80mgQ4W:TLD200mg | indrect | Low | Serious Imprecision |
| IXE80mgQ4W:UST45mgQ12W | indrect | Low | Serious Imprecision |
| IXE80mgQ4W:UST90mgQ12W | indrect | Low | Serious Imprecision |
| RIS150mg:SEC150mgQ4W | indrect | Low | Imprecision、Heterogeneity |
| RIS150mg:SEC300mgQ4W | indrect | Low | Imprecision、Heterogeneity |
| RIS150mg:TLD200mg | indrect | Low | Serious Imprecision |
| RIS150mg:UST45mgQ12W | indrect | Low | Serious Imprecision |
| RIS150mg:UST90mgQ12W | indrect | Low | Serious Imprecision |
| SEC150mgQ4W:TLD200mg | indrect | Low | Serious Imprecision |
| SEC150mgQ4W:UST45mgQ12W | indrect | Low | Serious Imprecision |
| SEC150mgQ4W:UST90mgQ12W | indrect | Low | Serious Imprecision |
| SEC300mgQ4W:TLD200mg | indrect | Low | Serious Imprecision |
| SEC300mgQ4W:UST45mgQ12W | indrect | Low | Serious Imprecision |
| SEC300mgQ4W:UST90mgQ12W | indrect | Low | Serious Imprecision |
| TLD200mg:UST45mgQ12W | indrect | Low | Serious Imprecision |
| TLD200mg:UST90mgQ12W | indrect | Low | Serious Imprecision |
| **MDA** | | | |
| BKZ160mgQ4W:PLACEBO | mixed | High |  |
| GUS100mgQ4W:GUS100mgQ8W | mixed | Low | Imprecision、Heterogeneity |
| GUS100mgQ4W:PLACEBO | mixed | High |  |
| GUS100mgQ8W:PLACEBO | mixed | High |  |
| IXE80mgQ2W:IXE80mgQ4W | mixed | Low | Serious Imprecision |
| IXE80mgQ2W:PLACEBO | mixed | High |  |
| IXE80mgQ4W:PLACEBO | mixed | High |  |
| PLACEBO:RIS150mg | mixed | High |  |
| PLACEBO:SEC150mgQ4W | mixed | High |  |
| PLACEBO:SEC300mgQ4W | mixed | High |  |
| PLACEBO:TLD200mg | mixed | High |  |
| SEC150mgQ4W:SEC300mgQ4W | mixed | Moderate | Imprecision |
| BKZ160mgQ4W:GUS100mgQ4W | indrect | Moderate | Heterogeneity |
| BKZ160mgQ4W:GUS100mgQ8W | indrect | High |  |
| BKZ160mgQ4W:IXE80mgQ2W | indrect | Low | Imprecision、Heterogeneity |
| BKZ160mgQ4W:IXE80mgQ4W | indrect | Low | Serious Imprecision |
| BKZ160mgQ4W:RIS150mg | indrect | High |  |
| BKZ160mgQ4W:SEC150mgQ4W | indrect | High |  |
| BKZ160mgQ4W:SEC300mgQ4W | indrect | Low | Imprecision、Heterogeneity |
| BKZ160mgQ4W:TLD200mg | indrect | Low | Serious Imprecision |
| GUS100mgQ4W:IXE80mgQ2W | indrect | Low | Serious Imprecision |
| GUS100mgQ4W:IXE80mgQ4W | indrect | Low | Serious Imprecision |
| GUS100mgQ4W:RIS150mg | indrect | Moderate | Heterogeneity |
| GUS100mgQ4W:SEC150mgQ4W | indrect | Low | Imprecision、Heterogeneity |
| GUS100mgQ4W:SEC300mgQ4W | indrect | Low | Serious Imprecision |
| GUS100mgQ4W:TLD200mg | indrect | Low | Serious Imprecision |
| GUS100mgQ8W:IXE80mgQ2W | indrect | Low | Serious Imprecision |
| GUS100mgQ8W:IXE80mgQ4W | indrect | Low | Serious Imprecision |
| GUS100mgQ8W:RIS150mg | indrect | Moderate | Imprecision |
| GUS100mgQ8W:SEC150mgQ4W | indrect | Low | Serious Imprecision |
| GUS100mgQ8W:SEC300mgQ4W | indrect | Low | Serious Imprecision |
| GUS100mgQ8W:TLD200mg | indrect | Low | Serious Imprecision |
| IXE80mgQ2W:RIS150mg | indrect | Low | Serious Imprecision |
| IXE80mgQ2W:SEC150mgQ4W | indrect | Low | Serious Imprecision |
| IXE80mgQ2W:SEC300mgQ4W | indrect | Low | Serious Imprecision |
| IXE80mgQ2W:TLD200mg | indrect | Low | Serious Imprecision |
| IXE80mgQ4W:RIS150mg | indrect | Moderate | Imprecision |
| IXE80mgQ4W:SEC150mgQ4W | indrect | Low | Serious Imprecision |
| IXE80mgQ4W:SEC300mgQ4W | indrect | Low | Serious Imprecision |
| IXE80mgQ4W:TLD200mg | indrect | Low | Serious Imprecision |
| RIS150mg:SEC150mgQ4W | indrect | Low | Imprecision、Heterogeneity |
| RIS150mg:SEC300mgQ4W | indrect | Moderate | Heterogeneity |
| RIS150mg:TLD200mg | indrect | Low | Serious Imprecision |
| SEC150mgQ4W:TLD200mg | indrect | Low | Serious Imprecision |
| SEC300mgQ4W:TLD200mg | indrect | Low | Serious Imprecision |
| **AEs** | | | |
| BKZ160mgQ4W:PLACEBO | mixed | High |  |
| BRO140mgQ2W:BRO210mg Q2W | mixed | Low | Imprecision、Heterogeneity |
| BRO140mg Q2W:PLACEBO | mixed | Low | Imprecision、Heterogeneity |
| BRO210mg Q2W:PLACEBO | mixed | Low | Serious Imprecision |
| GUS100mgQ4W:GUS100mg Q8W | mixed | Low | Imprecision、Heterogeneity |
| GUS100mg Q4W:PLACEBO | mixed | Low | Imprecision、Heterogeneity |
| GUS100mg Q8W:PLACEBO | mixed | Low | Serious Imprecision |
| IXE80mgQ2W:IXE80mgQ4W | mixed | Low | Serious Imprecision |
| IXE80mgQ2W:PLACEBO | mixed | Moderate | Imprecision |
| IXE80mgQ4W:PLACEBO | mixed | Low | Serious Imprecision |
| PLACEBO:RIS150mg | mixed | Moderate | Imprecision |
| PLACEBO:SEC150mgQ4W | mixed | Moderate | Imprecision |
| PLACEBO:SEC300mgQ4W | mixed | Moderate | Imprecision |
| PLACEBO:TLD200mg | mixed | Low | Serious Imprecision |
| PLACEBO:UST45mgQ12W | mixed | Moderate | Serious Imprecision |
| PLACEBO:UST90mgQ12W | mixed | Low | Imprecision、Heterogeneity |
| SEC150mgQ4W:SEC300mgQ4W | mixed | Low | Serious Imprecision |
| UST45mgQ12W:UST90mgQ12W | mixed | Low | Imprecision、Heterogeneity |
| BKZ160mgQ4W:BRO140mg Q2W | indrect | Moderate | Heterogeneity |
| BKZ160mgQ4W:BRO210mg Q2W | indrect | Moderate | Imprecision |
| BKZ160mgQ4W:GUS100mg Q4W | indrect | Moderate | Imprecision |
| BKZ160mgQ4W:GUS100mg Q8W | indrect | Moderate | Imprecision |
| BKZ160mgQ4W:IXE80mgQ2W | indrect | Low | Serious Imprecision |
| BKZ160mgQ4W:IXE80mgQ4W | indrect | Low | Serious Imprecision |
| BKZ160mgQ4W:RIS150mg | indrect | Moderate | Imprecision |
| BKZ160mgQ4W:SEC150mgQ4W | indrect | High |  |
| BKZ160mgQ4W:SEC300mgQ4W | indrect | High |  |
| BKZ160mgQ4W:TLD200mg | indrect | Moderate | Imprecision |
| BKZ160mgQ4W:UST45mgQ12W | indrect | Low | Serious Imprecision |
| BKZ160mgQ4W:UST90mgQ12W | indrect | Moderate | Imprecision |
| BRO140mgQ2W:GUS100mg Q4W | indrect | Low | Serious Imprecision |
| BRO140mgQ2W:GUS100mg Q8W | indrect | High |  |
| BRO140mg Q2W:IXE80mgQ2W | indrect | Moderate | Imprecision |
| BRO140mg Q2W:IXE80mgQ4W | indrect | Low | Serious Imprecision |
| BRO140mg Q2W:RIS150mg | indrect | Low | Serious Imprecision |
| BRO140mg Q2W:SEC150mgQ4W | indrect | Low | Serious Imprecision |
| BRO140mg Q2W:SEC300mgQ4W | indrect | Low | Serious Imprecision |
| BRO140mg Q2W:TLD200mg | indrect | Low | Serious Imprecision |
| BRO140mgQ2W:UST45mgQ12W | indrect | Moderate | Imprecision |
| BRO140mgQ2W:UST90mgQ12W | indrect | Low | Serious Imprecision |
| BRO210mgQ2W:GUS100mg Q4W | indrect | Low | Serious Imprecision |
| BRO210mgQ2W:GUS100mg Q8W | indrect | Low | Serious Imprecision |
| BRO210mg Q2W:IXE80mgQ2W | indrect | Low | Serious Imprecision |
| BRO210mg Q2W:IXE80mgQ4W | indrect | Low | Serious Imprecision |
| BRO210mg Q2W:RIS150mg | indrect | Low | Serious Imprecision |
| BRO210mg Q2W:SEC150mgQ4W | indrect | Low | Serious Imprecision |
| BRO210mg Q2W:SEC300mgQ4W | indrect | Low | Serious Imprecision |
| BRO210mg Q2W:TLD200mg | indrect | Low | Serious Imprecision |
| BRO210mg Q2W:UST45mgQ12W | indrect | Low | Serious Imprecision |
| BRO210mg Q2W:UST90mgQ12W | indrect | Low | Serious Imprecision |
| GUS100mg Q4W:IXE80mgQ2W | indrect | Low | Serious Imprecision |
| GUS100mg Q4W:IXE80mgQ4W | indrect | Low | Serious Imprecision |
| GUS100mg Q4W:RIS150mg | indrect | Low | Serious Imprecision |
| GUS100mg Q4W:SEC150mgQ4W | indrect | Moderate | Imprecision |
| GUS100mg Q4W:SEC300mgQ4W | indrect | Low | Imprecision、Heterogeneity |
| GUS100mg Q4W:TLD200mg | indrect | Low | Serious Imprecision |
| GUS100mgQ4W:UST45mgQ12W | indrect | Low | Serious Imprecision |
| GUS100mgQ4W:UST90mgQ12W | indrect | Low | Serious Imprecision |
| GUS100mg Q8W:IXE80mgQ2W | indrect | Low | Imprecision、Heterogeneity |
| GUS100mg Q8W:IXE80mgQ4W | indrect | Low | Serious Imprecision |
| GUS100mg Q8W:RIS150mg | indrect | Low | Serious Imprecision |
| GUS100mg Q8W:SEC150mgQ4W | indrect | Moderate | Imprecision |
| GUS100mg Q8W:SEC300mgQ4W | indrect | Low | Imprecision、Heterogeneity |
| GUS100mg Q8W:TLD200mg | indrect | Low | Serious Imprecision |
| GUS100mgQ8W:UST45mgQ12W | indrect | Low | Imprecision、Heterogeneity |
| GUS100mgQ8W:UST90mgQ12W | indrect | Low | Serious Imprecision |
| IXE80mgQ2W:RIS150mg | indrect | Low | Serious Imprecision |
| IXE80mgQ2W:SEC150mgQ4W | indrect | Moderate | Imprecision |
| IXE80mgQ2W:SEC300mgQ4W | indrect | Moderate | Imprecision |
| IXE80mgQ2W:TLD200mg | indrect | Low | Imprecision、Heterogeneity |
| IXE80mgQ2W:UST45mgQ12W | indrect | Low | Serious Imprecision |
| IXE80mgQ2W:UST90mgQ12W | indrect | Low | Serious Imprecision |
| IXE80mgQ4W:RIS150mg | indrect | Low | Serious Imprecision |
| IXE80mgQ4W:SEC150mgQ4W | indrect | Low | Serious Imprecision |
| IXE80mgQ4W:SEC300mgQ4W | indrect | Low | Serious Imprecision |
| IXE80mgQ4W:TLD200mg | indrect | Low | Serious Imprecision |
| IXE80mgQ4W:UST45mgQ12W | indrect | Low | Serious Imprecision |
| IXE80mgQ4W:UST90mgQ12W | indrect | Low | Serious Imprecision |
| RIS150mg:SEC150mgQ4W | indrect | Moderate | Imprecision |
| RIS150mg:SEC300mgQ4W | indrect | Moderate | Imprecision |
| RIS150mg:TLD200mg | indrect | Low | Serious Imprecision |
| RIS150mg:UST45mgQ12W | indrect | Low | Serious Imprecision |
| RIS150mg:UST90mgQ12W | indrect | Low | Serious Imprecision |
| **SAEs** | | | |
| BKZ160mgQ4W:PLACEBO | mixed | Low | Serious Imprecision |
| BRO140mg Q2W:BRO210mg Q2W | mixed | Low | Serious Imprecision |
| BRO140mg Q2W:PLACEBO | mixed | Low | Serious Imprecision |
| BRO210mg Q2W:PLACEBO | mixed | Low | Serious Imprecision |
| GUS100mg Q4W:GUS100mg Q8W | mixed | Low | Serious Imprecision |
| GUS100mg Q4W:PLACEBO | mixed | Low | Serious Imprecision |
| GUS100mg Q8W:PLACEBO | mixed | Low | Serious Imprecision |
| IXE80mgQ2W:IXE80mgQ4W | mixed | Low | Serious Imprecision |
| IXE80mgQ2W:PLACEBO | mixed | Low | Serious Imprecision |
| IXE80mgQ4W:PLACEBO | mixed | Low | Serious Imprecision |
| PLACEBO:RIS150mg | mixed | Low | Serious Imprecision |
| PLACEBO:SEC150mgQ4W | mixed | Low | Serious Imprecision |
| PLACEBO:SEC300mgQ4W | mixed | Low | Serious Imprecision |
| PLACEBO:TLD200mg | mixed | Low | Serious Imprecision |
| PLACEBO:UST45mgQ12W | mixed | Low | Serious Imprecision |
| PLACEBO:UST90mgQ12W | mixed | Low | Serious Imprecision |
| SEC150mgQ4W:SEC300mgQ4W | mixed | Low | Serious Imprecision |
| UST45mgQ12W:UST90mgQ12W | mixed | Low | Serious Imprecision |
| BKZ160mgQ4W:BRO140mg Q2W | indrect | Low | Serious Imprecision |
| BKZ160mgQ4W:BRO210mg Q2W | indrect | Low | Serious Imprecision |
| BKZ160mgQ4W:GUS100mg Q4W | indrect | Low | Serious Imprecision |
| BKZ160mgQ4W:GUS100mg Q8W | indrect | Low | Serious Imprecision |
| BKZ160mgQ4W:IXE80mgQ2W | indrect | Low | Serious Imprecision |
| BKZ160mgQ4W:IXE80mgQ4W | indrect | Low | Serious Imprecision |
| BKZ160mgQ4W:RIS150mg | indrect | Low | Serious Imprecision |
| BKZ160mgQ4W:SEC150mgQ4W | indrect | Low | Serious Imprecision |
| BKZ160mgQ4W:SEC300mgQ4W | indrect | Low | Serious Imprecision |
| BKZ160mgQ4W:TLD200mg | indrect | Low | Serious Imprecision |
| BKZ160mgQ4W:UST45mgQ12W | indrect | Low | Serious Imprecision |
| BKZ160mgQ4W:UST90mgQ12W | indrect | Low | Serious Imprecision |
| BRO140mg Q2W:GUS100mg Q4W | indrect | Low | Serious Imprecision |
| BRO140mg Q2W:GUS100mg Q8W | indrect | Low | Serious Imprecision |
| BRO140mg Q2W:IXE80mgQ2W | indrect | Low | Serious Imprecision |
| BRO140mg Q2W:IXE80mgQ4W | indrect | Low | Serious Imprecision |
| BRO140mg Q2W:RIS150mg | indrect | Low | Serious Imprecision |
| BRO140mg Q2W:SEC150mgQ4W | indrect | Low | Serious Imprecision |
| BRO140mg Q2W:SEC300mgQ4W | indrect | Low | Serious Imprecision |
| BRO140mg Q2W:TLD200mg | indrect | Low | Serious Imprecision |
| BRO140mg Q2W:UST45mgQ12W | indrect | Low | Serious Imprecision |
| BRO140mg Q2W:UST90mgQ12W | indrect | Low | Serious Imprecision |
| BRO210mg Q2W:GUS100mg Q4W | indrect | Low | Serious Imprecision |
| BRO210mg Q2W:GUS100mg Q8W | indrect | Low | Serious Imprecision |
| BRO210mg Q2W:IXE80mgQ2W | indrect | Low | Serious Imprecision |
| BRO210mg Q2W:IXE80mgQ4W | indrect | Low | Serious Imprecision |
| BRO210mg Q2W:RIS150mg | indrect | Low | Serious Imprecision |
| BRO210mg Q2W:SEC150mgQ4W | indrect | Low | Serious Imprecision |
| BRO210mg Q2W:SEC300mgQ4W | indrect | Low | Serious Imprecision |
| BRO210mg Q2W:TLD200mg | indrect | Low | Serious Imprecision |
| BRO210mgQ2W:UST45mgQ12W | indrect | Low | Serious Imprecision |
| BRO210mgQ2W:UST90mgQ12W | indrect | Low | Serious Imprecision |
| GUS100mg Q4W:IXE80mgQ2W | indrect | Low | Serious Imprecision |
| GUS100mg Q4W:IXE80mgQ4W | indrect | Low | Serious Imprecision |
| GUS100mg Q4W:RIS150mg | indrect | Low | Serious Imprecision |
| GUS100mg Q4W:SEC150mgQ4W | indrect | Low | Serious Imprecision |
| GUS100mg Q4W:SEC300mgQ4W | indrect | Low | Serious Imprecision |
| GUS100mg Q4W:TLD200mg | indrect | Low | Serious Imprecision |
| GUS100mgQ4W:UST45mgQ12W | indrect | Low | Serious Imprecision |
| GUS100mgQ4W:UST90mgQ12W | indrect | Low | Serious Imprecision |
| GUS100mg Q8W:IXE80mgQ2W | indrect | Low | Serious Imprecision |
| GUS100mg Q8W:IXE80mgQ4W | indrect | Low | Serious Imprecision |
| GUS100mg Q8W:RIS150mg | indrect | Low | Serious Imprecision |
| GUS100mg Q8W:SEC150mgQ4W | indrect | Low | Serious Imprecision |
| GUS100mg Q8W:SEC300mgQ4W | indrect | Low | Serious Imprecision |
| GUS100mg Q8W:TLD200mg | indrect | Low | Serious Imprecision |
| GUS100mgQ8W:UST45mgQ12W | indrect | Low | Serious Imprecision |
| GUS100mgQ8W:UST90mgQ12W | indrect | Low | Serious Imprecision |
| IXE80mgQ2W:RIS150mg | indrect | Low | Serious Imprecision |
| IXE80mgQ2W:SEC150mgQ4W | indrect | Low | Serious Imprecision |
| IXE80mgQ2W:SEC300mgQ4W | indrect | Low | Serious Imprecision |
| IXE80mgQ2W:TLD200mg | indrect | Low | Serious Imprecision |
| IXE80mgQ2W:UST45mgQ12W | indrect | Low | Serious Imprecision |
| IXE80mgQ2W:UST90mgQ12W | indrect | Low | Serious Imprecision |
| IXE80mgQ4W:RIS150mg | indrect | Low | Serious Imprecision |
| IXE80mgQ4W:SEC150mgQ4W | indrect | Low | Serious Imprecision |
| IXE80mgQ4W:SEC300mgQ4W | indrect | Low | Serious Imprecision |
| IXE80mgQ4W:TLD200mg | indrect | Low | Serious Imprecision |
| IXE80mgQ4W:UST45mgQ12W | indrect | Low | Serious Imprecision |
| IXE80mgQ4W:UST90mgQ12W | indrect | Low | Serious Imprecision |
| RIS150mg:SEC150mgQ4W | indrect | Low | Serious Imprecision |
| RIS150mg:SEC300mgQ4W | indrect | Low | Serious Imprecision |
| RIS150mg:TLD200mg | indrect | Low | Serious Imprecision |
| RIS150mg:UST45mgQ12W | indrect | Low | Serious Imprecision |
| RIS150mg:UST90mgQ12W | indrect | Low | Serious Imprecision |
| SEC150mgQ4W:TLD200mg | indrect | Low | Serious Imprecision |
| SEC150mgQ4W:UST45mgQ12W | indrect | Low | Serious Imprecision |
| SEC150mgQ4W:UST90mgQ12W | indrect | Low | Serious Imprecision |
| SEC300mgQ4W:TLD200mg | indrect | Low | Serious Imprecision |
| SEC300mgQ4W:UST45mgQ12W | indrect | Low | Serious Imprecision |
| SEC300mgQ4W:UST90mgQ12W | indrect | Low | Serious Imprecision |
| TLD200mg:UST45mgQ12W | indrect | Low | Serious Imprecision |
| TLD200mg:UST90mgQ12W | indrect | Low | Serious Imprecision |

ACR: American College of Rheumatology response; MDA: minimal disease activity; AEs: adverse events; SAEs: serious adverse events; PBO: PLACEBO; BKZ: bimekizumab; BRO: brodalumab; IXE: ixekizumab; SEC: secukinumab; TLD: tildrakizumab; RIS: risankizumab; GUS: guselkumab; UST: ustekinumab.

# **Supplementary Figure S3**: Confidence in evidence for all drugs compared to placebo

Confidence in evidence for all drugs to treat psoriatic arthritis compared to placebo according to CINeMA (Confidence in Network Meta-analysis). Five outcomes were considered. The bars present the percentage of outcomes with each evidence level (e.g. for BKZ160mgQ4W 83.3% of the reported outcomes had a high evidence level, 0% a moderate, and 17% a low). The drugs with the largest proportion of outcomes ranked with high certainty of evidence are presented on top (e.g. for BKZ160 mgQ4W 83% of the outcomes had a high level of evidence). As CINeMA does not consider comparisons for which no data are available, we added this information in the white bars (e.g. for UST 90mgQ12W for 67% of the outcomes no data were available at all). BKZ:bimekizumab; BRO:brodalumab; IXE:Ixekizumab; SEC:secukinumab; TLD:tildrakizumab; RIS:risankizumab; GUS:guselkumab; UST:ustekinumab. Colour code: green=high, blue=moderate, orange=low, red=very low, white=percentage of outcomes with no data available.

# **Supplementary Figure S4:Funnel plots of the efficacy and safety outcomes.**

A ACR20 B ACR50


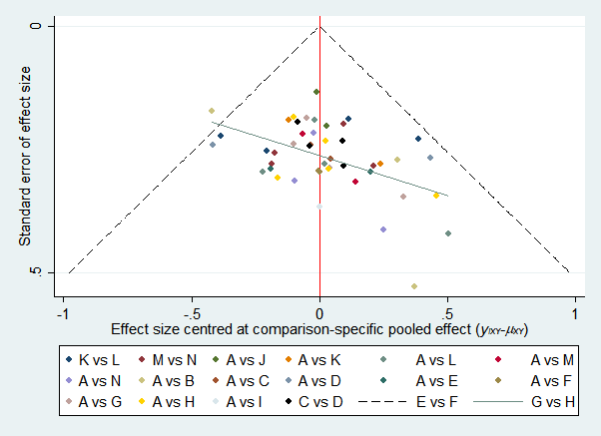

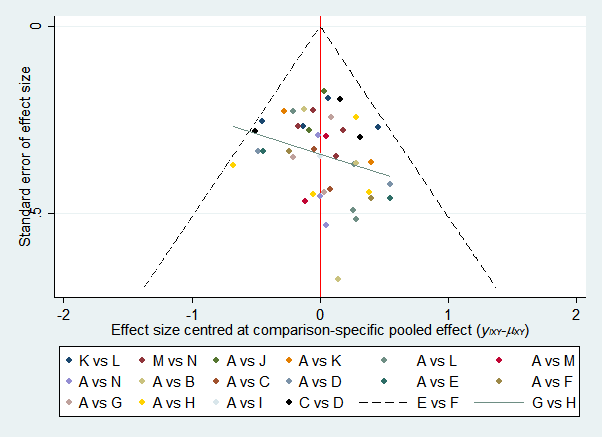


C ACR70 D AEs


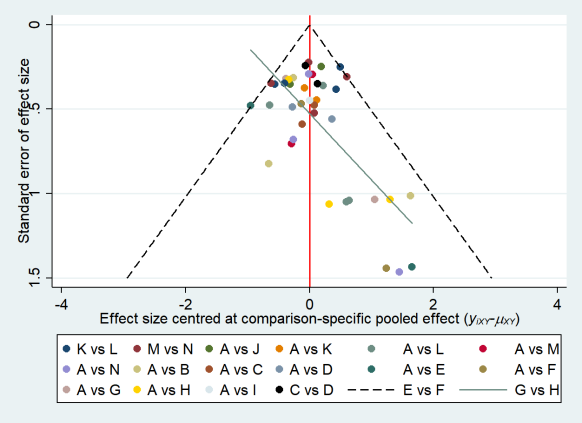

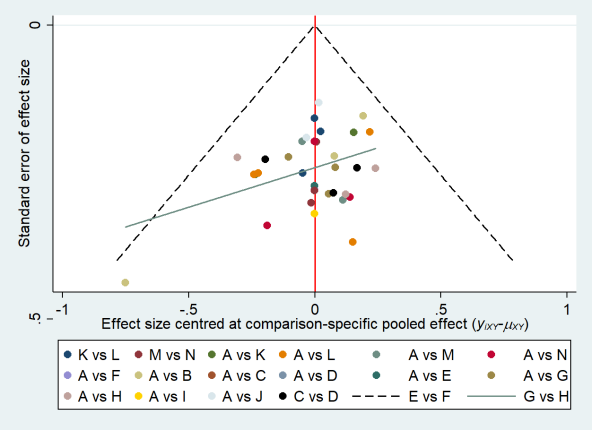


ACR:American College of Rheumatology response;MDA:minimal disease activity; AEs: adverse events; SAEs: serious adverse events;

# **Supplementary Figure S5: SUCRA of achieving ACR20 at 16 or 24 weeks in network meta-analysis.**


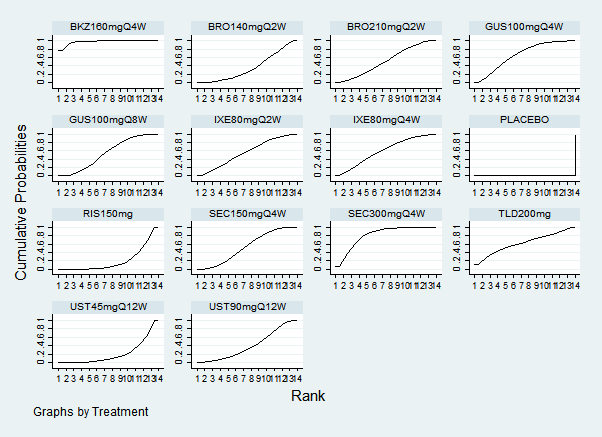


BKZ:bimekizumab; BRO:brodalumab; IXE:Ixekizumab; SEC:secukinumab; TLD:tildrakizumab; RIS:risankizumab; GUS:guselkumab; UST:ustekinumab

# **Supplementary Figure S6: SUCRA of achieving ACR20 at 12 or 24 weeks in network meta-analysis.**


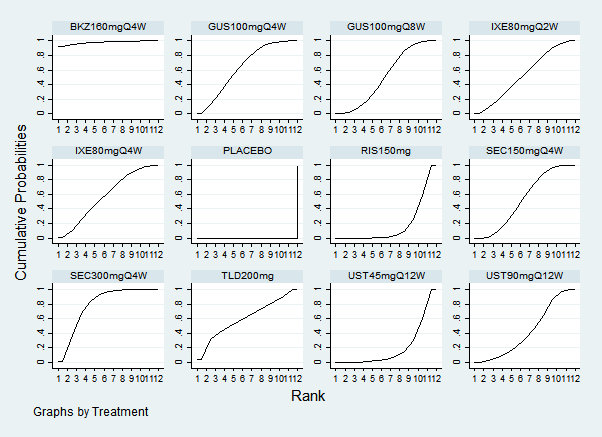


BKZ:bimekizumab; IXE:Ixekizumab; SEC:secukinumab; TLD:tildrakizumab; RIS:risankizumab; GUS:guselkumab; UST:ustekinumab

# **Supplementary Figure S7: SUCRA of achieving ACR20 at 12 or 16 weeks in network meta-analysis.**


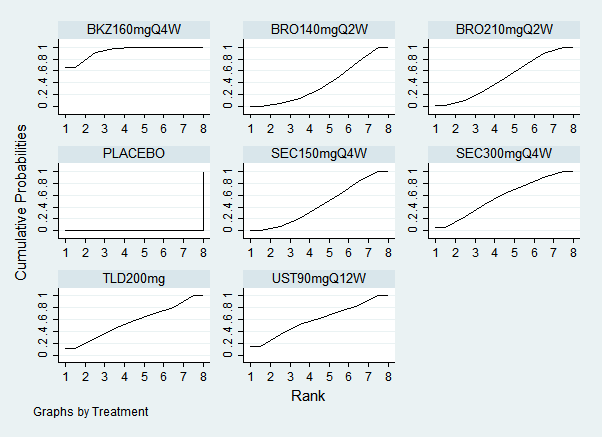


BKZ:bimekizumab; BRO:brodalumab; SEC:secukinumab; TLD:tildrakizumab; GUS:guselkumab; UST:ustekinumab
